# Smart Catheters for Diagnosis, Monitoring, and Therapy

**DOI:** 10.1002/adhm.202503913

**Published:** 2025-11-17

**Authors:** Azra Yaprak Tarman, Samiha Ahmed, Majed Othman Althumayri, Megan Guy, Darlenne Chavez Lugo, Frances S. Ligler, George T. Ligler, Rahmi Oklu, Hiroshi Kawahira, Michael J. McShane, Jun Kameoka, Jonathan Bova, Hatice Ceylan Koydemir

**Affiliations:** ^1^ Department of Biomedical Engineering Texas A&M University College Station Texas 77843 USA; ^2^ Center for Remote Health Technologies and Systems Texas A&M Engineering Experiment Station College Station Texas 77843 USA; ^3^ Department of Medical Equipment Technology College of Applied Medical Sciences Majmaah University Al Majmaah 11952 Saudi Arabia; ^4^ Department of Multidisciplinary Engineering Texas A&M University College Station Texas 77840 USA; ^5^ Laboratory for Patient Inspired Engineering Mayo Clinic Phoenix Arizona 85054 USA; ^6^ Department of Surgery Division of Gastroenterological General and Transplant Surgery Jichi Medical University 3311‐1, Yakushiji Shimotsuke‐shi Tochigi 329‐0498 Japan; ^7^ Medical Simulation Center Jichi Medical University 3311‐1, Yakushiji Shimotsuke‐shi Tochigi 329‐0498 Japan; ^8^ Department of Materials Science and Engineering Texas A&M University College Station Texas 77843 USA; ^9^ Graduate School of Fundamental Science and Engineering Information Production and System Research Waseda University Kitakyushu Fukuoka 808‐0135 Japan; ^10^ Comparative Medicine Program Division of Research Texas A&M University College Station TX 77843 USA

**Keywords:** catheter‐integrated biosensors, multimodal systems, on‐body monitoring, personalized healthcare, smart catheters, therapeutic systems

## Abstract

This review explores smart catheters as an emerging class of medical devices that combine embedded sensors, robotics, and communication systems with increasing functionality and complexity to enable real‐time health monitoring, diagnostics, and treatment. Evolving from traditional catheters used as drains or entry ports, smart systems are now able to track blood pressure, temperature, biochemical signals, and mechanical forces within the body with a high degree of accuracy. Advances in materials, wireless communication, and robotic navigation have helped reduce common risks like infection and catheter blockage while also improving precision catheter placement for minimally invasive procedures. This review highlights recent developments across a variety of different types of smart catheters, ranging from sensing and imaging tools to therapeutic and multimodal systems. Additionally, it discusses the challenges that remain, including biocompatibility, long‐term performance, and clinical translation. The incorporation of new capabilities is changing how catheters are used, and these new uses promise to enable more personalized and responsive healthcare.

## Graphical abstract

This study presents a comprehensive review of smart catheters, an emerging class of medical devices that integrate embedded sensors, robotics, and communication systems, offering increased functionality and complexity to enable real‐time health monitoring, diagnostics, and treatment.

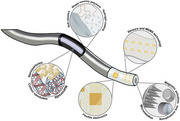

## 1 Introduction

Smart catheters, defined as catheter systems integrated with sensors, actuators, and/or communication technologies, have emerged as transformative tools in healthcare, significantly enhancing diagnostic capabilities, therapeutic efficacy, and patient monitoring.^[^ ^1^, ^2^ ^]^ This new generation of catheters builds on a long history of catheter insertion. Catheter utilization dates back as early as 3000 B.C., where initial designs served primarily for urinary retention management.^[^ ^3^ ^]^ Notably, the technological progression witnessed a significant leap in the 1930s when Dr. Frederic E.B. Foley introduced the latex balloon catheter, now universally known as the “Foley catheter”.^[^ ^4^ ^]^ Today, urinary catheters remain indispensable in medical practices globally. In the United States alone, approximately 30 million urinary catheters are utilized annually, with roughly 20% of hospitalized patients requiring their use at any given time.^[^ ^5^ ^]^ Globally, more than 100 million urinary catheters are deployed annually, underscoring their substantial clinical relevance and extensive utility across medical disciplines.

**Figure 1:**
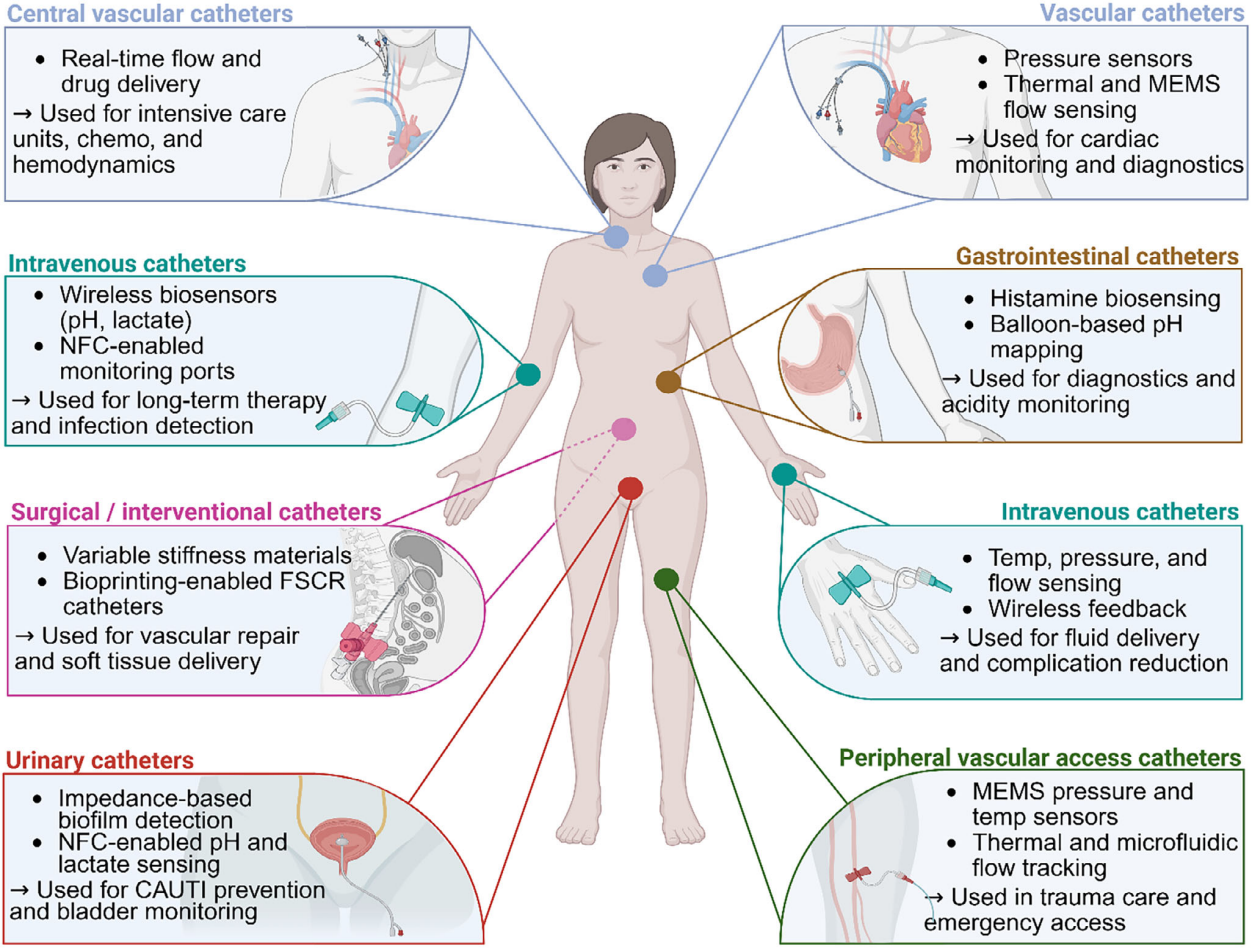
Anatomical distribution of smart catheter applications. Key catheterization sites are shown with associated sensing, therapeutic, and interventional functions. The schematic highlights implantable and insertable platforms for pressure, flow, biochemical sensing, wireless communication, and robotic capabilities across urinary, vascular, gastrointestinal, intravenous, and surgical systems. Created in BioRender.

Beyond urinary applications, vascular catheters are also extensively employed, playing an important role in healthcare interventions. These include central venous catheters (CVCs), peripheral intravenous catheters, arterial catheters, pulmonary artery catheters, and hemodialysis catheters, among others.^[^ ^6^ ^]^ Specifically, CVCs are fundamental elements in diverse clinical interventions, including intensive care management, surgical procedures, chemotherapy, and hematological therapies, with over five million insertions annually in the United States (US) alone.^[^ ^7^ ^]^ Peripheral intravenous catheters are similarly integral clinically, with their use in therapeutic administration having become the most common invasive procedure in acute care hospitals. Their insertion into an estimated 80% of patients at some point during hospitalization corresponds to more than one billion intravenous placements globally each year.^[^ ^7^ ^]^ A schematic overview of key anatomical sites and associated smart catheter applications is presented in **Figure** **1** to contextualize the spatial and functional diversity discussed throughout this review.

Despite their widespread utilization, traditional catheters present several major limitations and associated risks. Foremost among these concerns is the heightened risk of catheter‐associated infections, including most prominently catheter‐associated urinary tract infections (CAUTIs) and catheter‐related bloodstream infections, which are among the leading healthcare‐associated infections worldwide. These infections contribute significantly to patient morbidity and mortality and substantially elevate healthcare expenses. Additionally, traditional catheter designs are susceptible to frequent blockages. Encrustation, biofilm formation, accumulation of tissue fragments, and blood clotting within the catheter lumen necessitate frequent catheter replacements and heightened complication risks.^[^ ^8^, ^9^ ^]^ Long‐term use of traditional catheters can also result in chronic tissue damage, such as vascular injuries or urethral strictures, which further exacerbates patient discomfort and healthcare burdens.^[^ ^10^ ^]^

Emerging smart catheter technologies address these significant challenges by incorporating features such as cutting‐edge sensor platforms,^[^ ^2^ ^]^ microelectronics,^[^ ^1^, ^11^ ^]^ advanced materials,^[^ ^12^, ^13^ ^]^ wireless communication modalities,^[^ ^14^, ^15^ ^]^ and controlled therapeutic delivery systems^[^ ^16^, ^17^ ^]^ to augment traditional catheter capabilities significantly. By leveraging real‐time monitoring of physiological and biochemical parameters—including pressure, temperature, pH, glucose, lactate, and oxygen levels—smart catheters facilitate continuous patient monitoring and prompt early detection of complications.^[^ ^11^, ^18^, ^19^ ^]^ Biosensor‐enabled smart catheters can provide continuous biochemical feedback, allowing healthcare providers to intervene swiftly and proactively when abnormalities are detected.^[^ ^20^, ^21^, ^22^, ^23^ ^]^ Integrated microsensors extend the catheter's diagnostic capabilities by providing real‐time measurements of physical parameters such as strain, flow, and pressure, essential for precise therapeutic management.^[^ ^2^, ^11^ ^]^ In addition, robotic catheter systems with magnetic and electromagnetic navigation capabilities have transformed minimally invasive procedures, enabling precise and controlled maneuverability of the catheter through complex vasculatures and anatomical structures. These robotic‐assisted catheters significantly reduce procedural invasiveness, expedite patient recovery, and minimize risks associated with large surgical incisions.^[^ ^24^ ^]^ Moreover, smart catheter systems can proactively mitigate the prevalent issue of catheter blockage through integrated drug‐eluting or anticoagulant‐release features.^[^ ^25^ ^]^

Despite substantial advancements in catheter development, several significant technical, clinical, and regulatory challenges persist, necessitating ongoing research and innovation. Long‐term biocompatibility and device durability remain prominent concerns in certain applications, as catheters used for extended periods must withstand prolonged physiological stresses without eliciting adverse tissue responses such as inflammation, thrombosis, or biofilm formation.^[^ ^26^ ^]^ Thus, comprehensive and stringent preclinical and clinical validations are necessary to ensure efficacy and patient safety, further complicating the already significant task of navigating intricate regulatory approval pathways for the integration of novel technologies into clinical practice.^[^ ^27^ ^]^

This review comprehensively discusses the recent advances in smart catheter technologies, emphasizing their diverse applications in diagnostics, therapeutic interventions, and minimally invasive surgeries. We examine commercially available and emerging catheter technologies focusing on sensor integration, wireless communication systems, advanced materials, and robotic enhancements that improve catheter functionality, precision, and patient outcomes. Additionally, we outline key challenges such as infection control, integration of multifunctional sensing platforms, and scalability issues to provide a holistic perspective on the status and future potential of smart catheters. In synthesizing these advances, this review highlights the evolving landscape of smart catheter technologies and their critical role in reshaping healthcare practices.

## 2 Sensor‐Integrated Smart Catheters

Sensor‐integrated catheters often incorporate state‐of‐the‐art sensor technology to measure and report physiological parameters like blood flow, pressure, or electrical activity in the heart over extended periods. By providing real‐time data, diagnostic catheters offer invaluable insights that can significantly impact patient outcomes and facilitate rapid response to emerging issues. Newer developments in fabrication techniques have enabled the design of highly sensitive, flexible, and biocompatible sensors suitable for intravascular environments. One major engineering challenge remaining is the seamless integration of these sensors onto the curvilinear and dynamic surfaces of catheter shafts. To address this, researchers are investigating materials with unique electrical and mechanical properties that are not only biocompatible and stretchable but also capable of maintaining sensor performance under bending or flexing. Innovations in material science and fabrication strategies are key to transforming conventional catheters into smart diagnostic tools.^[^ ^28^, ^29^ ^]^ **Table** **1** provides a summary of various smart sensing catheters, highlighting their sensing modalities, integration strategies, and features that advance the catheters. In the following paragraphs, we will discuss the different types of sensor‐integrated catheters based on the various physiological parameters measured.

**Table 1:** Summaries of Recent Sensor‐integrated Catheters.

Sensing Category	Bioanalyte/Parameter	Sensing Mechanism	Smart Feature	Integration with Catheter	Reference
Pressure‐sensing	Intravascular blood pressure	Piezoelectric sensing using BaTiO_3_‐PDMS nanocomposite	Targeted piezoelectric response through aligned composite; conformal integration; high transducing performance	Sensor placed on the proximal end of a special catheter provided with electrical connections	[34]
	Intraluminal pressure and arterial pulse	Piezoelectric sensing using P(VDF‐TrFE)	Miniaturization and selective integration for site‐specific pressure mapping	Sensors laterally inserted into laser‐cut notches on metallic catheter	[35]
	Upper airway soft tissue pressure	Piezoresistive sensors	360° mapping of airway pressure for spatial localization	Sensors fabricated by direct femtosecond laser 3D engraving on catheter surface	[36]
Force‐sensing	Tip force in x, y, z axes during electrosurgery	3 axially aligned FBG sensors	3D force sensing with machine learning‐based force reconstruction	Two‐point pasting method to integrate FBGs circumferentially around a soft polymer catheter	[39]
	Catheter tip contact force during ablation	Piezoresistive sensing using doped silicon bridges	Ring MEMS architecture for radial force uniformity and real‐time ablation feedback	Sensor embedded in customized ablation tip using force transfer structures and direct contact pillars	[47]
	Catheter tip contact force during ablation and haptic feedback	MEMS piezoresistive bridge sensor	Vibro‐haptic feedback for real‐time tactile force perception	Sensor mounted at the base of a customized catheter tip via threaded acrylic coupler	[48]
	Tip contact force in all 3 axes	Multi‐core FBG sensors embedded in helical spring	Multi‐axis FBG sensing enabling 3D contact force reconstruction	FBG‐inscribed multi‐core fiber fixed at both ends inside a nitinol catheter using adhesive	[49]
	Biomechanical contact force (surgical navigation)	Optical fiber‐based force sensing via Fabry‐Perot cavity	In‐situ force sensing with real‐time feedback; closed‐loop magnetic control	Coaxial integration of optical fiber sensor and ring magnet with silicone catheter via ultraviolet (UV adhesive and fusion bonding	[45]
Temperature‐sensing	Local temperature at implantation site (e.g., brain)	Ionic thermoresistive sensing in hydrogel	Real‐time local temperature sensing; Self‐healing hydrogel with ionic conductivity; wireless readout	Hydrogel polymerized in situ onto plasma‐treated catheter surface	[53]
	Intracranial pressure and brain temperature in traumatic brain injury	MEMS piezoresistive pressure sensor with switchable temperature mode	Dual‐mode sensing via single MEMS sensor	MEMS sensor embedded in flexible PCB; catheter formed by rolling the flexible circuit through a funnel	[54]
	Intracranial temperature monitoring	Thin‐film resistance temperature detector (Au‐based, four‐wire configuration)	Spiral‐wrapped flexible electronics with a resolution higher than 0.1°	Sensor spirally rolled around a rod and inserted into catheter lumen and post‐treated	[55]
Flow‐monitoring	Breathing direction and rate	Thermal flow sensing with flow direction sensors	Dual‐function flow rate and direction sensing with temperature compensation	Flexible polyimide sensor film inserted and fixed in catheter using heat‐shrink tubing	[58]
	Blood flow, tissue contact, ablation performance	Thermal flow sensing via thermistors and microheater	Thermal and electrical dual‐function balloon for hemodynamic feedback; conformal integration	Sensors and electrodes with serpentine geometry bonded onto balloon catheter via conformal wrapping	[59]
	Tracheal airflow rate, obstruction detection	MEMS thermal flow sensing via microheater and temperature sensor	Steerable catheter with shape memory actuators; real‐time flow mapping with segmental resolution	Sensor dies wire‐bonded to flexible PCB and embedded in catheter body using PDMS molding	[60]
Biomarker‐sensing	Interstitial pH and lactate for infection/biofilm	Voltammetric (pH) and amperometric (lactate) sensing via needle type electrochemical sensor	Batteryless, wireless biomarker sensing with smartphone‐based NFC readout	Miniature needle electrodes inserted into totally implanted access port structure; encapsulated with PDMS	[23]
	Bacterial growth and biofilm formation	Impedimetric sensing using interdigitated electrodes	Wireless, dual‐function system for real‐time bacterial detection and biofilm treatment via bioelectric effect	Flexible electrodes embedded in a 3D‐printed insert module interfaced with the inner lumen of a commercial Foley catheter	[62]
	Intestinal histamine for irritable bowel syndrome diagnosis	Non‐faradaic impedance spectroscopy with MIP‐coated electrodes	Real‐time histamine detection in intestinal fluid environment	Electrodes embedded inside catheter tip using polyether ether ketone centering piece and PDMS sealing	[63]
	Cortical electrical activity	Low‐noise electrical recording via soft electrodes	Soft electronics for low‐noise electro‐encephalogram in dynamic cerebrospinal fluid environment	Electrode array transferred via water‐soluble tape onto PDMS, then wrapped and bonded onto catheter with plasma activation	[64]
	Inflammatory marker (C‐reactive protein)	Organic field‐effect transistor ‐based sensing	Ultrathin sensor with flexible biointerface and low‐voltage CRP detection	Transferred to catheter using water floatation method and bonded via vertical interconnect access and silver paste	[65]
	Cardiac tissue surface pH	Potentiometric sensing via stretchable gold electrodes electroplated with iridium oxide	Multiplexed, real‐time pH mapping on dynamically moving tissue surfaces	Electrodes transferred on to balloon catheter surface using water‐soluble tape and bonded with silicone adhesive	[66]
Multi‐sensing	Cardiac contact pressure, tissue temperature, ECG	Resistive and electrical sensing via multilayer sensor array	Multiplexed mapping of pressure, temperature, and electrograms with therapeutic actuation	Multilayer sensor array transfer printed onto balloon catheter	[19]
	Contact force and thermal mapping	FBG‐based optical sensing	Thermal control and multi‐parameter sensing in variable stiffness catheter	FBG array embedded in biopsy needle and fixed inside catheter lumen using thermal paste and cyanoacrylate adhesive	[68]
	Urinary pH, bladder pressure, internal temperature	Potentiometric (pH), piezoresistive (pressure), and thermistor‐based (temperature) sensing	Multiparameter sensing with flexible hybrid circuits	Sensors embedded into recesses of custom 3D‐printed catheter tip and encapsulated with polyurethane resin	[69]

### 2.1 Pressure‐Sensing Catheters

Pressure monitoring is crucial for diagnosing and managing cardiovascular diseases, including coronary artery stenosis, in‐stent restenosis, and thrombosis.^[^ ^30^ ^]^ Abnormal pressure gradients across vascular structures, such as stenotic lesions or thrombotic blockages, are key indicators of disease severity and progression.^[^ ^31^ ^]^ There is a need for directly accessing internal physiological environments for providing real‐time pressure monitoring, which is often unattainable through external indirect measurements. Smart catheters equipped with pressure‐sensing technologies have emerged as a promising minimally invasive solution to address challenges associated with inaccuracies in indirect pressure measurements, imprecision due to limited spatial densities, and a lack of continuous monitoring capabilities in current pressure monitoring methodologies. Recent advancements have leveraged piezoelectric, piezoresistive, and capacitive sensing mechanisms, coupled with biocompatible and flexible materials, for pressure sensing in the vasculature.^[^ ^32^, ^33^ ^]^ We now focus on emerging technologies driving advancements in smart catheter‐based pressure sensing.

Among the various approaches developed to increase accuracy in dynamic blood pressure monitoring, D'Ambrogio et al.^[^ ^34^ ^]^ introduced a vascular catheter‐integrated piezoelectric sensor utilizing barium‐titanate/polydimethylsiloxane (BaTiO_3_‐PDMS) nanocomposites for fractional flow reserve measurements, providing real‐time, precise pressure monitoring to evaluate the severity of coronary artery stenosis. The sensor^[^ ^34^ ^]^ measures dynamic blood pressure by converting arterial pressure variations into electrical signals via the piezoelectric properties of the structured BaTiO_3_‐PDMS composite. This structured configuration improves directional sensitivity, enabling the detection of minute pressure changes during dynamic cardiac cycles. Using a cardiac simulation arm, the device achieved clinically relevant sensitivity (0.75 mVkPa^−1^) and operated within the physiological pressure range typical of coronary arteries during in‐vitro testing. The sensor's biocompatibility, flexibility, and low‐cost fabrication make it a promising alternative to conventional sensors, which are limited by stiffness, complex circuitry, and high material costs.

**Figure 2:**
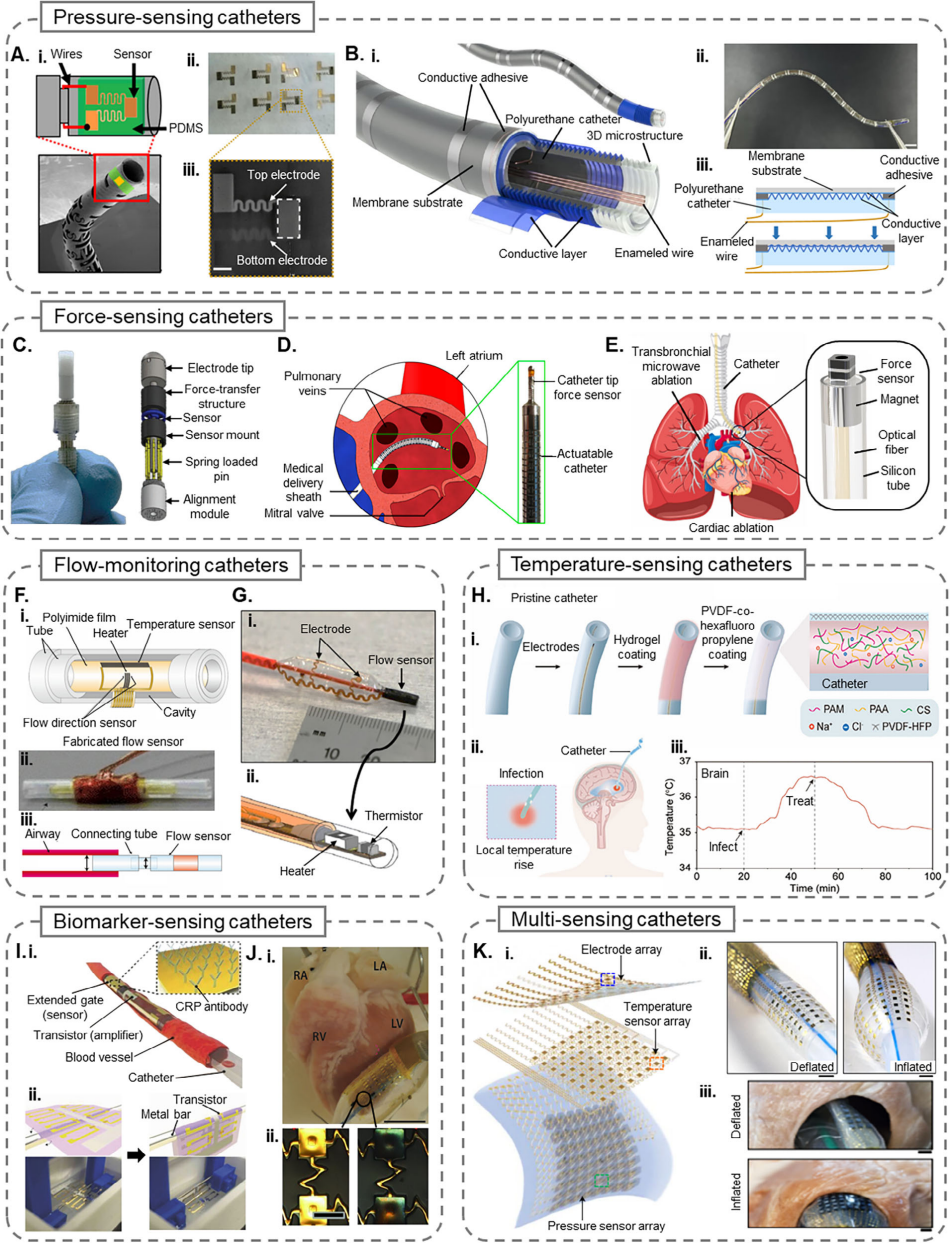
A i) A concept illustration of a medical catheter equipped with an integrated pressure sensor, designed for vascular and surgical procedures. ii) Multiple pressure sensors fabricated together on a piezoelectric film substrate, shown prior to individual separation. iii) Scanning electron microscopy (SEM) image highlighting the structure of a single pressure sensor (scale bar = 500 µm). Reprinted with permission from ref. [35] B i) Diagram depicting a flexible multilayer pressure sensor array designed for catheter integration. ii) Photograph showing the flexible catheter‐mounted sensing array (scale bar = 5 mm) iii) Cross‐sectional schematic of a sensor unit illustrating its configuration under no pressure (top) and applied pressure (bottom). Reprinted with permission from ref. [36] C) Image of a customized catheter tip integrated with a MEMS‐based force sensor within a protective packaging unit.^[^ ^48^ ^]^ D) Illustration of a catheter‐based cardiac ablation procedure targeting the left atrium, with a zoomed view showing the force‐sensing catheter tip and its actuation mechanism. Reprinted with permission from ref. [49] E) Surgical use scenarios of a magnetically actuated catheter, highlighting its application in transbronchial microwave ablation of lung nodules and AF treatment. The inset showcases the catheter design with an embedded force sensor and magnetic ring. Reprinted with permission from ref. [45]. F i) Schematic representation of the catheter‐based flow sensor configuration. ii. Photograph of a fabricated flow sensor integrated onto a catheter system. iii. Illustration of the catheter‐based flow sensor inserted into a rat's airway using a connecting tube. Reproduced with permission from ref. [58] from Springer Nature. G) Optical image of a 3D balloon catheter incorporating both flow sensors and electrodes, with an exploded schematic detailing the sensor placement near the balloon tip. Reproduced with permission from ref. [59]. H i) Schematic showing a hydrogel‐coated temperature‐sensing catheter design using poly‐ (vinylidene fluoride‐co‐hexafluoropropylene) composites. ii) Illustration describing how catheter implantation can trigger localized brain infections detectable by temperature rise. iii) Graph depicting brain temperature fluctuations during infection onset and treatment phases. Reproduced with permission from ref. [58] 2023 Wiley‐VCH GmbH. I i) Conceptual depiction of a ventricular catheter embedded with a CRP biosensor for inflammatory marker detection within blood vessels. ii. Ultrathin organic field‐effect transistor sensor in its flat state (floating in water) and the sensor in its bent state, lifted by a metal bar, demonstrating its flexibility and transferability to a curvy surface. Reprinted with permission from ref. [65] 2018 WILEY‐VCH Verlag GmbH & Co. KGaA, Weinheim. J i) Ex vivo image of a rabbit heart with an implanted balloon catheter, exposing the pH‐sensing electrodes (scale bar = 1 cm). ii. Magnified images showing the gold electrodes before (left) and after (right) electroplating with iridium oxide (scale bar = 0.5 mm). Reprinted with permission from ref. [66] 2014 WILEY‐VCH Verlag GmbH & Co. KGaA, Weinheim. K i) Schematic of a multilayer catheter device integrating arrays for electrophysiological mapping, temperature sensing, and pressure measurement. ii) Images demonstrating the transferred electrode array onto a silicone balloon catheter, shown in both inflated and deflated conditions (scale bar = 2 mm). iii) Application of a balloon catheter embedded with 3D pressure sensors within a pig heart model (scale bar = 2 mm). Reprinted with permission from ref. [19]

Another example of pressure monitoring using a piezoelectric sensor is the polyvinylidene‐fluoride‐trifluoroethane (PVDF‐TrFE) based sensor designed by Gil et al.,^[^ ^35^ ^]^ which enables accurate intraluminal and subcutaneous pressure monitoring via a catheter. The sensor converts mechanical force into electrical signals using the piezoelectric effect of the PVDF‐TrFE layer sandwiched between gold electrodes, as shown in **Figure** **2**A. The sensor exhibits a high signal responsivity of 0.63 µVPa^−1^ and maintains stability over 400000 loading cycles under physiological pressure (≈12 kPa), with a tested operational range up to 40 kPa. The sensor supports bilateral pressure sensing and provides real‐time pressure data when integrated into a bronchoscopy catheter. This capability enhances surgical precision during minimally invasive procedures by offering intraoperative feedback and further allows clinicians to track physiological changes during post‐operative recovery, enabling more effective patient monitoring and outcome assessment.

Continuous, site‐specific monitoring of upper airway pressure is necessary for accurately diagnosing and managing obstructive sleep apnea (OSA). Shang et al.^[^ ^36^ ^]^ introduced a flexible, sensor‐integrated catheter featuring a high‐resolution pressure‐sensing array designed to map soft tissue pressure across the upper airway, enabling precise localization of obstruction events during sleep. Unlike traditional catheter types, this device is inserted through the nasal cavity to monitor the upper airways. As shown in Figure 2B, the system uses a polyurethane catheter base micro‐structured via femtosecond laser engraving, coated with conductive poly (3,4‐ethylenedioxythiphene) polystyrene sulfonate, and encapsulated in polydimethylsiloxane (PDMS) to form a piezoresistive sensing array. With ten sensing units distributed over 6 cm, the device demonstrated a peak sensitivity of 38.1 ΩmmHg^−1^, fast response (660 ms), and minimal signal degradation across 200 cycles. Implanted in a porcine OSA model, the sensor array successfully identified and localized airway obstructions consistent with computed tomography and polysomnography data, including multi‐site collapses. Due to its high flexibility (bending modulus 5.86 MPa) and biocompatibility, this smart catheter has the potential for long‐term, minimally invasive deployment in OSA diagnostics.

Senthil Kumar et al.^[^ ^37^ ^]^ introduced a stretchable capacitive pressure‐sensing sleeve designed to wrap around commercial Foley catheter balloons, enabling direct and continuous intra‐abdominal pressure monitoring at the site of interest. The sensor uses a micro‐structured dielectric layer composed of Ecoflex and cyanoethyl pullulan, patterned using abrasive paper templating to enhance compressibility and sensitivity. Silver nanowire‐based flexible electrodes were layered onto the dielectric and encapsulated in a diaphragm to form a robust, conformal sensor structure. The capacitive sensor demonstrated two distinct linear pressure sensitivity regions (0–1 kPa and 1–5 kPa) with a response time of about 500 ms. Deployed on a 14 French (Fr) Foley catheter — a standard clinical size referring to the catheter's outer diameter —the device was tested in a bladder pressure simulation chamber, where it accurately tracked dynamic pressure changes in response to intravesical pressure fluctuations. This sensing sleeve also shows strong potential for wireless integration and broader application in procedures such as resuscitative endovascular balloon occlusion of the aorta and endoscopic renal pressure monitoring.^[^ ^37^ ^]^

### 2.2 Force‐Sensing Catheters

Precise control and measurement of contact force are essential in catheter‐based interventions where tissue manipulation, lesion formation, or navigation is involved, such as cardiac ablation, endoscopic dissection, and bronchial procedures.^[^ ^38^, ^39^ ^]^ Inadequate force application can result in suboptimal catheter positioning or insufficient tissue interaction, which may compromise treatment effectiveness, while exertion of excessive force risks tissue damage, perforation, or procedural failure.^[^ ^40^ ^]^ Traditional catheters typically lack integrated force feedback, requiring clinicians to rely on indirect cues or experience‐based estimation and compromising safety and efficacy.^[^ ^41^ ^]^ The challenge lies in embedding force sensors into highly miniaturized, flexible, and biocompatible catheter tips without compromising steerability or real‐time responsiveness. Recent advancements in catheter‐based force sensing have focused on the conformability of the sensor on a curvy surface, such as that of a catheter.^[^ ^42^ ^]^ Technologies like piezoresistive micro‐electromechanical systems (MEMS),^[^ ^43^ ^]^ optical fiber‐based sensors like fiber Bragg gratings (FBGs),^[^ ^44^ ^]^ and soft robotic technology^[^ ^45^, ^46^ ^]^ are enabling precise, real‐time, multi‐axis force detection with high spatial resolution. The following examples^[^ ^39^, ^45^, ^47^, ^48^, ^49^ ^]^ illustrate how such technologies improve catheter‐based interventions across cardiovascular, gastrointestinal, and respiratory domains.

Sitaramgupta et al.^[^ ^47^ ^]^ explored piezoresistive MEMS‐based force sensors integrated into CVCs to facilitate ablation procedures. One design featured a suspended‐bridge piezoresistive sensor with four orthogonal bridges embedded with piezo resistors. Integrated into a 3.5 mm catheter tip, the sensor achieved a normalized sensitivity of ≈0.022 N^−1^ over a 0–0.35 N range. One of the crucial features of this smart catheter is the vibrotactile haptic handle coupled with it, which allows clinicians to perceive different force ranges through fingertip feedback. Validation on porcine heart tissue confirmed the system's reliability in providing proprioceptive tactile cues, bridging the gap between sensor data and user perception. In a later study, Sitaramgupta et al.^[^ ^48^ ^]^ introduced a ring‐shaped MEMS sensor, as shown in Figure 2C, with a dual‐resistor configuration per bridge, offering enhanced directional sensitivity and irrigation compatibility. With a per‐bridge force range of 0.3 N and an integration‐ready form factor, the sensor delivered accurate real‐time measurements during ex vivo cardiac ablation.

The presence of precise force sensing at the catheter tip is a leading factor in determining minimally invasive surgical outcomes, where forces below or above the optimal range (0.1–0.4 N) may lead to incomplete ablation or tissue perforation, respectively.^[^ ^50^ ^]^ Al‐Ahmad et al.^[^ ^49^ ^]^ developed a compact, 3D force sensor using a multi‐core optical fiber with inscribed FBGs, embedded in a helical spring structure to enable longitudinal and lateral force measurements. The sensor achieves decoupled sensing via differential strain detection across FBG cores and employs two unstrained FBGs for real‐time temperature compensation, enabling high accuracy even under thermal fluctuation. With a miniaturized profile (2.2 mm diameter, 16.3 mm length), the device demonstrated a longitudinal force resolution of 7.4 mN and a lateral force resolution of 0.8 mN. Integrated into a pneumatically actuated surgical catheter, the FBG‐multi core fiber sensor provides a means for force‐guided ablation, enabling shape sensing and distributed force monitoring along the catheter body. An illustration of the catheter performing ablation in the left atrium is shown in Figure 2D.

Expanding the scope of catheter‐integrated FBG sensors to gastrointestinal procedures, Ben Hassen et al.^[^ ^39^ ^]^ introduced a tri‐axial force‐sensing catheter for endoscopic submucosal dissection. The device integrates three FBGs into a multi‐lumen polymer catheter using a two‐point bonding method with nitinol reinforcement for improved strain transfer. Calibrated across 30 spatial directions, the system captures axial and transverse forces up to ±500 mN and utilizes a hybrid data‐driven model for real‐time 3D force reconstruction. With <3% root mean square error in lateral and <10% in axial forces, the catheter offers reliable tactile feedback, facilitating safer and more controlled dissection procedures.

For effective control of contact force during interventional surgeries, Li et al.^[^ ^45^ ^]^ developed a small‐scale magnetic soft robotic catheter integrating an optical fiber‐based Fabry–Pérot force sensor and a coaxial ring‐shaped permanent magnet for active steering and real‐time force feedback (Figure Figure 2E). The microfabricated sensor, designed to detect the reactive force that is equal and opposite to the force applied, achieved a sensitivity of 0.69 nmkPa^−1^ and a linear force range of up to 1 N, suitable for applications like cardiac ablation. Integrated into a 2.5 mm diameter silicone catheter, the system demonstrated precise navigation and force‐controlled tissue interaction in bronchus phantoms and ex vivo pig organs. The feedback loop mechanism enabled stable force application within clinically relevant ranges (0.1–0.4 N), demonstrating its suitability for precise force control and directional navigation during minimally invasive procedures.

### 2.3 Flow‐Monitoring Catheters

Monitoring airflow, blood flow, and hemodynamic parameters is essential for diagnosing and managing various medical conditions, from vascular diseases to airway obstructions and cerebral injuries.^[^ ^51^, ^52^ ^]^ Conventional catheter systems often lack integrated flow‐sensing capabilities or rely on bulky, rigid components that limit flexibility and continuous monitoring. Challenges lie in miniaturizing sensors for compatibility with narrow anatomical structures, achieving directional sensitivity in dynamic fluid environments, and maintaining sensor stability during catheter deformation or high‐pressure conditions. Researchers are developing innovative smart catheter technologies to enable real‐time, localized, and accurate flow measurements, even under the mechanical stresses of complex anatomical navigation.

Hasegawa et al.^[^ ^53^ ^]^ improved a catheter‐based thermal flow sensor for bidirectional airflow sensing, which is necessary for respiratory applications involving reciprocating flow. The authors focused on miniaturization by combining a single temperature compensation element and two flow direction sensors, reducing device area significantly compared to prior designs. The sensor was fabricated using thin‐film metal deposition and packaged in a dual‐layer catheter tube with integrated thermal isolation, as shown in Figure 2F. The flow‐monitoring smart catheter measured both flow and directionality in a rat model, capturing physiological breathing parameters and heart activity. This optimized design reduced power consumption and area requirements for improving compatibility with small‐diameter airways.

Klinker et al.^[^ ^54^ ^]^ developed a balloon catheter integrated with stretchable electronics capable of simultaneously performing electrical stimulation, ablation, and monitoring blood flow. As shown in Figure 2G, the flow sensor consists of a thermistor‐heater pair configured on a compliant balloon substrate and designed to maintain a constant temperature using a Proportional‐Integral‐Derivative controlled thermal feedback loop. Blood flow is then determined from the heater power required to maintain thermal equilibrium. The system includes a reference thermistor for temperature drift compensation and enables volumetric flow rate computation in real‐time. The catheter was validated in porcine models, showing compatibility with high‐pressure inflation (≈2 atm) and vascular geometries. The integration of soft, bioresorbable electronics and flow sensors within a stent enables real‐time blood flow monitoring during angioplasty‐relevant procedures. In vivo demonstration in a porcine femoral artery model was used to prove the sensor's functionality under physiologically relevant conditions, supporting its utility during and after endovascular interventions such as angioplasty.

Alekya et al.^[^ ^55^ ^]^ introduced an intubation catheter equipped with MEMS‐based thermal flow sensors and antagonistic actuators composed of shape memory alloys for characterizing airflow patterns in stenosed tracheas. The system employs four platinum‐based microheaters integrated on a flexible printed circuit board (PCB) and encapsulated in PDMS. The sensors provide localized air velocity measurements, with 2.4 times rise in flow rate near 50% tracheal obstruction, showing that the system is sensitive to early‐stage stenosis. The springs, composed of the shape memory alloy and integrated into the catheter, provided directional steering within the complex airway. Validated using stenosis models in excised sheep tracheas, this system presents a minimally invasive approach to quantify airway obstructions and can be utilized in central airway obstruction management.

### 2.4 Temperature‐Sensing Catheters

Another important physiological parameter to monitor is the local temperature of internal organs. Accurate temperature monitoring can facilitate the detection of infections, enable tracking of intracranial temperature in traumatic brain injuries, and prevent post‐surgical complications.^[^ ^56^, ^57^ ^]^ However, conventional temperature monitoring approaches often rely on external probes or discrete measurements, lacking spatial resolution and precision. Challenges persist in developing sensors that conform to soft tissues and can maintain high sensitivity and stability in fluidic or mechanically dynamic environments. Recent works^[^ ^58^, ^59^, ^60^ ^]^ have introduced catheter‐integrated temperature sensors based on hydrogel composites, MEMS, and thin‐film resistance temperature detectors, offering improved biocompatibility, mechanical compliance, and precision.

For example, Li et al.^[^ ^58^ ^]^ introduced a temperature‐sensing hydrogel coating, shown in Figure Figure 2H, to enable real‐time infection detection when integrated onto various medical catheters, including peripheral venous catheters, urinary catheters, nasogastric tubes, and ventricular drainage tubes. With a temperature coefficient of resistance of 2.90% °C^−1^, the hydrogel, a poly(vinylidene fluoride‐co‐hexafluoropropylene) covered stable poly (acrylamide/acrylic acid/chitosan) triple network, is mechanically compatible with soft tissues, minimizing the risk of damage as well as inflammation. The hydrogel demonstrated stability under prolonged exposure and deformation in simulated cerebrospinal fluid. In a rat brain model, the use of this hydrogel led to a 90% survival rate by enabling detection of infection‐related temperature changes and facilitating timely intervention, such as administration of antibiotics through the hydrogel‐coated catheter.

Bao et al.^[^ ^59^ ^]^ designed a smart catheter system incorporating MEMS‐based pressure and temperature sensors for real‐time monitoring in the management of traumatic brain injury. This flexible ventricular catheter integrates sensors on a biocompatible flexible substrate, transmitting data to an external system for visualization and analysis. The catheter achieves a root mean square error of ±1.5 mmHg for pressure and ±0.08 °C for temperature, with a sampling rate of 10 Hz. Validated in laboratory animals, the system shows promise for reducing complications and improving patient outcomes through minimally invasive multimodal monitoring.

Similarly, another group^[^ ^60^ ^]^ reported a smart ventricular catheter that incorporates a thin‐film resistance temperature detector for intracranial monitoring in patients suffering traumatic brain injury. The resistance temperature detector achieved a sensitivity of 67 mV°C^−1^ with minimal drift, demonstrating performance comparable to commercial probes in vitro.

### 2.5 Biomarker‐Sensing Catheters

Real‐time monitoring of biochemicals as well as biophysical parameters is paramount in identifying complications during and after surgery and for effectively managing chronic diseases.^[^ ^61^ ^]^ Unlike conventional catheter systems that primarily provide mechanical access or fluid drainage, smart biosensing catheters integrate sensors to continuously monitor key biomarkers such as pH, lactate, inflammatory proteins, histamine, and oxygen saturation. Recent advances^[^ ^23^, ^62^, ^63^, ^64^, ^65^, ^66^ ^]^ have addressed challenges with conformability, sensor stability, and sensitivity by integrating technologies such as flexible electronics, stretchable materials, organic semiconductors, and molecularly selective coatings into catheter‐based biosensors.

In the field of oncology, for instance, early changes in pH and lactate levels can serve as indicators of biofilm formation in totally implanted access ports; these ports are commonly used in cancer patients who require long‐term central venous access for chemotherapy. To address this challenge, Gil et al.^[^ ^23^ ^]^ developed a near‐field‐communication (NFC)‐enabled, needle‐implanted catheter designed for real‐time pH and lactate monitoring. This enables early detection of biofilms. The sensor enables wireless, battery‐less monitoring of these markers through a smartphone interface, and the sensor seamlessly integrates into existing implanted access ports, eliminating the need for traditional blood analysis. Detection accuracy and performance were validated through in vitro experiments, including breast phantom modeling.^[^ ^23^ ^]^ While the system effectively enables wireless data transmission and demonstrates good sensitivity under physiological conditions, its long‐term functionality has not been evaluated. Since the lactate biosensor relies on immobilized lactate oxidase, an enzyme known to degrade over time, it might limit the sensor's operational lifespan. Given this, there is a need for in‐vivo studies that assess how rapidly the sensing performance deteriorates, especially under continuous or repeated use.

In a related effort to address the complications of biofilm formation in long‐term access devices, Huiszoon et al.^[^ ^62^ ^]^ addressed CAUTIs by modifying a Foley catheter with flexible interdigitated electrodes for biofilm detection and treatment. Integrated into a 3D‐printed insert, the electrodes enable impedance‐based detection of *E. coli* colonization and administer a bioelectric treatment by applying low‐voltage alternating current fields to enhance bacterial susceptibility to antibiotics. The Bluetooth‐enabled control system and smartphone interface enable wireless operation, providing real‐time feedback and monitoring the antimicrobial response while preserving standard catheter drainage and balloon functions. However, extended in vivo studies are necessary for clinical applications.

Wackers et al.^[^ ^63^ ^]^ addressed the challenge of diagnosing irritable bowel syndrome by creating a catheter‐based impedimetric sensor for histamine detection in the duodenum, which is a biomarker of mast cell activation. The system includes four titanium wires embedded in a silicone catheter tip having an outer diameter of 5 mm. Two of the catheters are coated with acrylic‐acid‐based molecularly imprinted polymers (MIP), which form histamine‐specific binding cavities, while two non‐imprinted wires serve as an on‐board reference. Histamine binding blocks ionic pathways inside the porous MIP layer, which leads to a measurable change in impedance. Housed within a gastrointestinal catheter with an integrated aspiration channel and protective cap, the sensor detected histamine concentrations between 5 and 200 nM in spiked duodenal fluids. While the prototype showed promising performance in short‐term in‐vitro and ex‐vivo tests, challenges remain in improving the mechanical durability of the MIP coating under peristaltic motion and ensuring reliable in vivo functionality under prolonged exposure to digestive enzymes. Moreover, the authors aim to reduce the outer diameter of the catheter in the future for patient comfort, making the device suitable for clinical deployment.

For neurosurgical applications, Yi et al.^[^ ^64^ ^]^ developed a flexible, low‐profile external ventricular drain catheter to reduce complications during hydrocephalus treatment by measuring local field potential. The catheter integrates soft‐embedded Ti/Au electrodes into a PDMS‐based membrane to measure electrical activity in the brain. The device has an elastic modulus of about 2.29 MPa, making it conformable to delicate neural tissues without disrupting ventricular drain functions. The integrated sensors provided real‐time feedback during in vitro and hydrogel phantom tests, showing the potential of the device for use in acute hydrocephalus monitoring and intracranial pressure monitoring. Despite successful in‐vitro validation, further in vivo tests must be performed to ensure long‐term durability of the sensors and to verify whether the local field potential recordings remain free of motion artefacts in the dynamic cerebrospinal fluid.

Additionally, Ji et al.^[^ ^65^ ^]^ developed an ultrathin biosensor‐integrated catheter using an organic field‐effect transistor capable of detecting C‐reactive protein (CRP), a key inflammatory marker monitored during surgery. Shown in Figure Figure 2I, the device features a 630 nm‐thick hybrid substrate composed of polyacrylonitrile and CYTOP fluoropolymer, encapsulating a thermally stable semiconductor and an anti‐CRP‐functionalized extended gate. The sensor was designed to withstand boiling water and steam sterilization and maintained stable electrical performance after 5000 bending cycles. The sensor was transferred onto a ventricular catheter via a water‐floatation technique. The sensor‐integrated catheter successfully distinguished CRP levels in serum samples, validating its application for intraoperative inflammation tracking, especially during cardiopulmonary bypass. While tested in serum only, the study demonstrates a clear foundation for future translation to whole blood.

To overcome the challenges of monitoring metabolic changes on the heart's dynamic, curvilinear surface, Chung et al.^[^ ^66^ ^]^ developed stretchable, multiplexed pH sensors integrated onto the surface of balloon catheters (Figure Figure 2J). Particularly during procedures such as cardiac catheterization or ablation, localized monitoring of pH is necessary to evaluate myocardial viability. In order to ensure conformal contact with cardiac tissue and to enable spatiotemporal mapping of pH, the authors used iridium oxide electrodes electroplated onto ultrathin elastomer membranes and configured either on balloon catheters or as conformable sheet arrays. These electrodes function as potentiometric sensors, where the open circuit potential between the working electrode and a reference electrode changes proportionally with the local hydrogen ion concentration. In an ex vivo study, the system successfully monitored spatiotemporal pH changes during ischemia‐reperfusion protocols on rabbit and human hearts.

Another example of physiological mapping using a catheter system is a balloon catheter developed by Xue et al.^[^ ^67^ ^]^ that integrates 3D electrodes for electrophysiological mapping of the colon. The flexible polyimide‐based electrode array, featuring hemispherical gold‐silver coated electrodes, was conformally integrated onto the surface of a pediatric balloon catheter using a modular wrapping and fixation strategy that ensures spatial alignment and contact stability. By inflating the balloon, the device establishes reliable electrical coupling with the mucosal and smooth muscle layers, maintaining signal quality despite intestinal motion. In vivo rabbit studies demonstrated the catheter's ability to capture multiphasic electrophysiological signals such as spike bursts, periodic slow waves, and pacemaker‐like rhythms. The authors demonstrated that the signal‐to‐noise ratio of the signals increases in its non‐planar form compared to planar arrays.

Monitoring regional oxygen saturation is critical during and after cardiothoracic procedures. However, conventional fiber‐optic catheter oximeters are limited by mechanical rigidity and tethered data acquisition systems. To overcome these limitations, Lu et al.^[^ ^21^ ^]^ developed a wireless, miniaturized oximetry system based on a soft, flexible catheter‐type probe integrated with high‐performance optoelectronics. The device consists of a 1.5 mm‐diameter encapsulated optical probe housing red (645 nm) and near‐infrared (950 nm) light‐emitting diodes and a photodiode, interfaced with a Bluetooth‐enabled skin‐mounted control module for data processing and wireless transmission. In‐vitro comparisons with commercial Swan‐Ganz catheters demonstrated comparable performance (i.e., R^2^ = 0.979). The catheter‐based oximeter was validated in vivo using a rat model. The in‐vivo studies showed that the device reliably captured cardiac oxygenation, heart rate, and respiratory activity with sub‐degree thermal rise. It also exhibited excellent mechanical compliance, substantially greater than that of conventional catheters, enabling conformal contact and safe, long‐term integration with biological tissue. Encapsulation in medical‐grade silicone provided stability under cyclic deformation and 8‐week implantation without adverse tissue response.

### 2.6 Multi‐Sensing Catheters

Multimodal sensing catheters represent a significant advancement in the development of smart medical devices, offering the ability to simultaneously monitor multiple physiological, biochemical, and mechanical parameters in real‐time.^[^ ^2^ ^]^ This integration is crucial in complex clinical scenarios, where relying on a single type of measurement can lead to an incomplete understanding of the patient's condition. However, aiming to add multimodal functionality in catheter‐based systems introduces new challenges, like maintaining device miniaturization and flexibility and avoiding cross‐interference between sensing modalities. Recent innovations^[^ ^19^, ^68^, ^69^ ^]^ have leveraged flexible electronics, hybrid material systems, and integrated wireless platforms to overcome many of these challenges, enabling catheters to have multiplexed sensing capabilities.

Han et al.^[^ ^19^ ^]^ developed a soft, multilayer balloon catheter system incorporating arrays of electronic sensors and actuators for multiplexed spatiotemporal mapping during cardiac surgery. As shown in Figure Figure 2K , the system includes layered arrays of electrodes for electrophysiological recording and stimulation, resistive temperature sensors, and 3D pressure sensors with strain gauge‐based transduction. The devices, fabricated using semiconductor microfabrication and transfer printing techniques, are mounted on elastomeric substrates and demonstrate high mechanical compliance (>30% stretchability), robust signal uniformity, and low hysteresis during biaxial deformation. The catheter supports programmable radiofrequency (RF) ablation, irreversible electroporation, and thermal mapping and has been validated in Langendorff‐perfused rabbit and human hearts. This platform exemplifies a high‐density, multimodal system capable of closed‐loop diagnostics and therapy delivery on soft tissue surfaces.

A multi‐sensing system embedded in a variable stiffness catheter was designed by De Tommasi et al.^[^ ^68^ ^]^ for simultaneous temperature and force sensing. The catheter, constructed from shape memory polymers, softens upon heating via an integrated Joule coil and is magnetically steerable for remote actuation. Five multiplexed FBGs embedded in an inner biopsy needle capture temperature change during thermal activation (up to 68 °C), while a sixth FBG located near the tip quantifies insertion force with high spatial resolution. The system effectively distinguished soft and stiff synthetic tissues, demonstrating its utility for force feedback in dynamic surgical environments. This approach addresses key limitations in tactile perception during minimally invasive surgery while enabling thermomechanical control of shape memory polymer behavior.

Focusing on urinary health, Dou et al.^[^ ^69^ ^]^ presented a reusable smart urinary catheter prototype for intermittent catheterization, particularly for those with spinal cord injury or disease —who often require frequent catheterization. The catheter integrates commercially available thermistors, pressure sensors, and a custom‐fabricated potentiometric pH sensor based on screen‐printed Prussian blue and Ag/AgCl electrodes. The sensors were encapsulated using biocompatible polyurethane coatings on 3D‐printed thermoplastic polyurethane catheter tips. The flexible hybrid electronics platform enables point‐of‐care data acquisition of bladder temperature, pressure, and urine pH, offering high accuracy and reliability even after multiple sterilization cycles. This system aims for early CAUTI detection and longitudinal urological health tracking by providing temperature, pressure, and pH information.

## 3 Interventional Smart Catheters

Smart technologies are being integrated into interventional or functional catheters to facilitate complex procedures like localized therapeutic intervention, sustained site‐specific drug delivery, minimally invasive surgeries, and high‐resolution imaging. Unlike traditional catheters, which rely heavily on external manipulation and fluoroscopic guidance, smart interventional catheters incorporate advanced actuation and control mechanisms. Magnetic steering, shape‐memory alloys, pneumatic and hydraulic systems, and electroactive polymers are some examples of newer technologies that allow for the catheter's enhanced maneuverability and functional precision within the body's complex anatomical structures. **Table** **2** provides a summary of various smart interventional catheters, highlighting their actuation mechanism, control, and integration strategies. In the following paragraphs, we will discuss the different types of interventional smart catheters based on the various functions they perform.

**Table 2:** Summaries of Recent Interventional Catheters.

Interventional Category	Primary Function	Control/Actuation Method	Smart Feature	Integration with Catheter	Reference
Minimally‐invasive surgical	Cardiac ablation	Magnetic steering and Joule heating‐induced stiffness change in conductive shape memory polymer	Monolithic catheter segments that simultaneously act as heater, temperature sensor, and variable stiffness actuator	Dip‐coated conductive shape memory polymer threads mounted with magnets and encapsulated on catheter shaft	[71]
	Ophthalmic microsurgery	Magnetic steering + Joule heating‐induced stiffness tuning in low melting point alloys	Phase‐change controlled stiffness (soft to rigid); real‐time feedback	Phase‐change alloy embedded via heating channels in polymer core	[72]
	Neurovascular access	Hydraulic actuation through 4 microfluidic channels for tip steering	3D steerable tip; guidewire‐free; soft hydraulics	Soft body hydraulics enclosed in flexible elastomer tubing	[73]
	Cardiac ablation	Hydraulic actuation using soft hydraulic filament artificial muscles	Combines variable stiffness stabilizing mechanism with soft force sensing and omnidirectional extension for stable cardiac ablation	Soft sensor embedded at catheter tip; VSSM integrated via cable‐driven lantern deployment and thermal phase change material stiffening	[74]
	Neuroembolization	Lorentz force‐based steering using quad‐configuration microcoils	Power‐optimized electromagnetic steering with MRI guidance; reduced tip heating	Microcoil array micromachined and wrapped around catheter tip	[75]
	Lumen defect sealing	Electrical activation of adhesive via retractable electrodes	On‐demand adhesive activation; retractable electrodes; deployable bioelectronic patch	Graphene patch bonded via balloon inflation and electrical contact	[76]
	Passive, steerable navigation through vasculature using flow‐driven propulsion.	Flow‐driven propulsion with magnetic tip steering.	Autonomous navigation of flexible microprobe through tortuous vasculature	Magnetic elastomer head bonded to polyimide based flexible microelectronic probe using epoxy	[79]
	Cardiac ablation	Pulsed field ablation with low‐voltage microelectrode discharge	Fractal microelectrodes for selective ablation at low voltage along with recording ECG and detecting contact force on tissue	Fractal microelectrodes bonded to catheter using hot‐pressed anisotropic conductive film and wire‐guiding via 3D printed molds	[83]
Therapeutic	Targeted delivery, manipulation, magnetic navigation	Self‐rolling of polymer‐electronics into Swiss‐roll ISACs	First self‐assembled microcatheter enabling multifunctional interventions.	Photolithographically patterned electronic layers rolled into Swiss‐roll microtubes via self‐assembly of hydrogel‐actuated polymer stacks	[11]
	In‐vivo capture and elimination of circulating tumor cells	Electroporation triggered by low‐voltage pulses applied through integrated liquid metal electrodes	Circulating tumor cell specific capture via antibody functionalized nanofiber + electroporation‐based cell killing	Electrospun nano fibers wrapped, and liquid metal‐polymer conductor coated over catheter core	[91]
	Tissue manipulation, biopsy, palpation	Magnetic actuation using external field and multi‐segmented magnets embedded in the tip	Compact FBG‐based triaxial force sensor integrated at the tip with multifunctional capabilities	Custom tip fabricated with three dedicated channels; components inserted and bonded using silicone adhesive	[93]
	Blood clot clearance in intraventricular hemorrhage treatment	Magnetic actuation of integrated microscale actuators using external time‐varying magnetic fields	Self‐clearing magnetic catheter that breaks down hematoma	Microactuators microfabricated and inserted into catheter lumen near inlet pore	[94]
	In‐vivo minimally invasive bioprinting	Remote magnetic actuation + internal pressurization for ink delivery	Bioprinting on curved internal tissues through a small incision using a programmable magnetoactive nozzle.	FSCR body injection‐molded from PDMS and hard magnetic particle composite with embedded polyactide fiber mesh	[95]
Imaging	Simultaneous IVUS + NIRF imaging of inflammation	Co‐registered NIRF and IVUS with rotational drive	Miniaturized hybrid 1.0 mm NIRF‐IVUS catheter	Lens and transducer inserted sequentially in 1.0 mm catheter lumen	[101]
	Non‐fluoroscopic endovascular navigation	Electric field generation + impedance mapping to vessel tree model	Electric‐fish‐inspired impedance‐based navigation	Electrode rings slid and bonded circumferentially to catheter tip	[102]
	Photoacoustic imaging of lipid‐laden plaques	Rotational scanning with laser pulse excitation + integrated ultrasound	Collinear design for optical/acoustic overlap and deep tissue penetration	Optical fiber and ultrasonic transducer aligned collinearly in 3D‐printed housing with rod mirror for radial imaging; assembled into 1.6 mm catheter	[103]
Drug‐eluting	Endovascular drug delivery	Balloon inflation	Microneedle‐enhanced tissue penetration for prolonged drug diffusion	Conformal transfer molding of MNs onto balloon surface using UV‐curable resin and flexible PDMS mold	[105]
	Sustained paclitaxel release post‐angioplasty	Near‐infrared laser‐induced phase‐change triggering for drug tip detachment	Photothermally triggered tip‐separable drug MNs on balloon surface	Conformal transfer molding of MNs onto balloon surface using UV‐curable resin and flexible PDMS mold	[106]
	Intra‐tissue sensing, ablation, and drug delivery	Manual insertion or spring‐loaded ejection	11‐function core‐shell microneedle catheter with biochemical, electrical, and therapeutic functions	Vertically aligned MN arrays embedded in hollow shells and integrated onto polyimide circuits along catheter shaft	[107]

### 3.1 Minimally Invasive Surgical Catheters

Surgical catheters are flexible, tubular medical devices used in numerous surgical intervention procedures, such as cardiovascular, urological, and neurological surgeries, to access specific areas within the body, including blood vessels and internal organs.^[^ ^70^ ^]^ Employed for guiding surgical instruments, maintaining tissue, and facilitating draining during surgeries, among countless other uses, these catheters are vital tools for minimally invasive surgeries. In equipping surgical catheters with sensors for monitoring physiological parameters, like pressure, temperature, or flow, surgical catheters can combine the flexibility and precision of traditional catheters with robotic technology, allowing for more controlled and accurate navigation within the complex anatomical structures, while maintaining biocompatibility.^[^ ^24^, ^71^ ^]^

**Figure 3:**
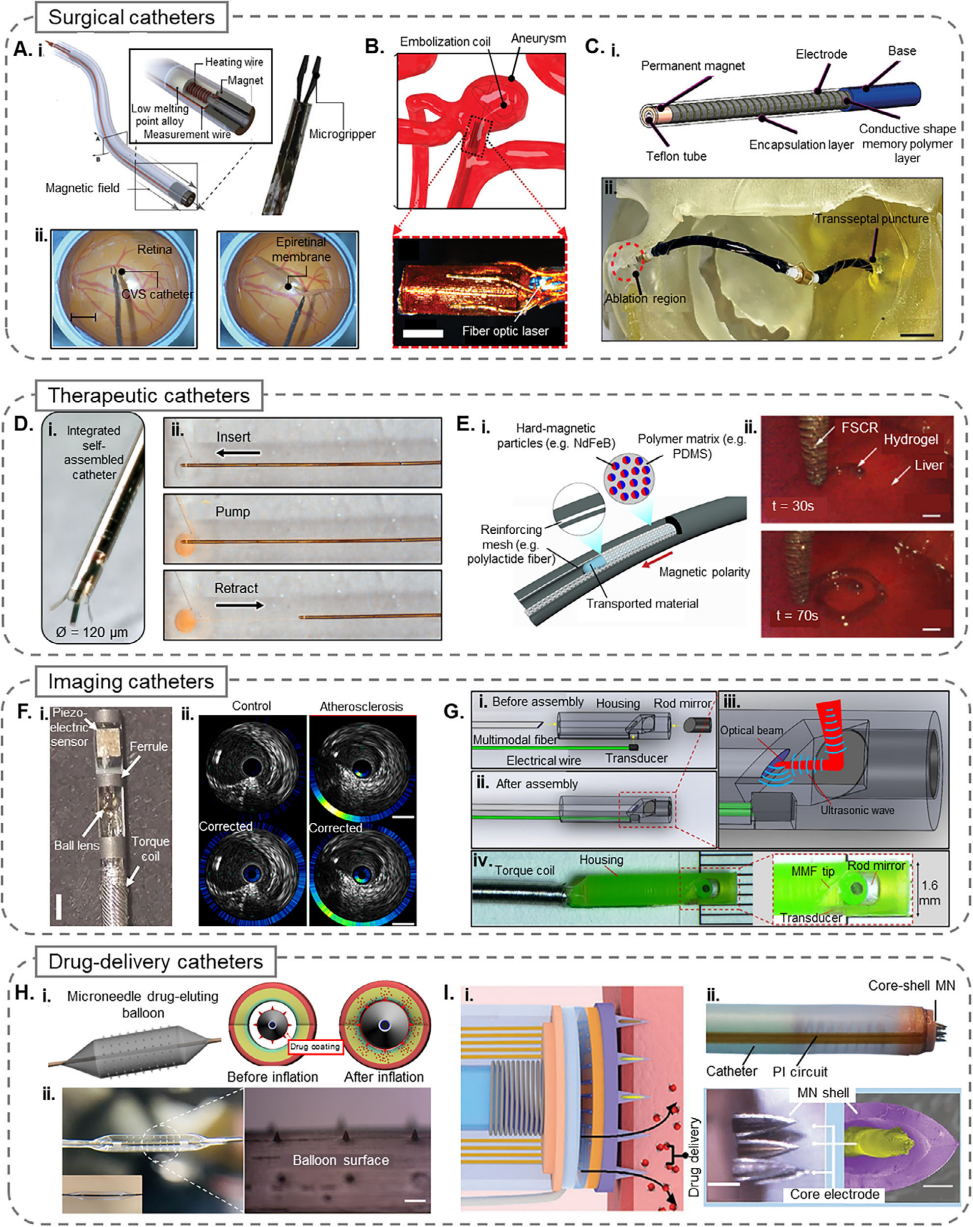
A i) Schematic and close‐up view of a continuous variable stiffness catheter, featuring an end‐effector tip embedded with a permanent magnet for magnetic steering. Heating wires adjust the phase of a low‐melting‐point alloy to modulate tip stiffness. ii) Robotic epiretinal membrane peeling on an eye phantom model, where the catheter's adjustable stiffness enables controlled opening and closing of a microgripper for precise membrane removal (scale bar = 5 mm). Reprinted with permission from ref. [72] 2021 The Authors. Advanced Science is published by Wiley‐VCH GmbH. B) Quad‐coil configuration device deploying an embolization coil to treat aneurysms, where coil deployment is tracked by MRI via image distortion caused by Lorentz actuation. Reprinted with permission from ref. [75] 2022 The Authors. Advanced Science is published by Wiley‐VCH GmbH. C i) Design of a single‐segment catheter incorporating a variable stiffness thread, a permanent magnet, and an electrode wrapped around a conductive shape memory polymer layer. ii) Depiction of the catheter traversing a transseptal puncture to reach the left atrium for cardiac ablation therapy (scale bar = 10 mm). Reprinted with permission from ref. [71] 2022 The Authors. Advanced Functional Materials published by Wiley‐VCH GmbH. D i) Micrograph of an ISAC tip with a diameter of ≈120 µm. ii. Sequential images showing ISAC navigation through a model channel and the delivery of a dyed model fluid to a target cavity simulating an aneurysm. Reprinted with permission from ref. [11]. Copyright 2021, The American Association for the Advancement of Science. E i) Illustration of a FSCR, composed of a polymer matrix embedded with hard‐magnetic particles and reinforced by a polylactide mesh. ii) Photographs demonstrating minimally invasive in vivo bioprinting of a conductive hydrogel on the liver surface of a rat over time (Scale bar = 1 mm). Reprinted with permission from ref. [95] Copyright 2021, The Author(s). F i) Design of a NIRF‐IVUS imaging catheter equipped with a piezoelectric sensor. ii) Hybrid NIRF‐IVUS in vivo imaging of a swine atherosclerosis model before and after distance correction (Scale bar: 1 mm). Reprinted with permission from ref. [101] 2021 Wiley‐VCH GmbH. G i) Main components of the collinear intravascular photoacoustic catheter prior to assembly. Ii) Fully assembled probe. iii) Close‐up of the catheter tip showing collinear alignment of optical and acoustic paths. iv. Image of the fabricated 1.6 mm diameter probe with inset detailing the tip structure. Reprinted with permission from ref. [103] Copyright 2016, The Authors. H i) Schematic of a microneedle drug‐eluting balloon, where microneedles coated with therapeutic agents on the balloon surface facilitate enhanced endovascular drug delivery. ii) Stereo‐ and optical microscopy images of the MNDEB surface, with an inset showing the pre‐transfer molded balloon surface (scale bar = 300 µm). Reprinted with permission from ref. [105] 2020 Elsevier B.V. I i) Schematic showing a microneedle‐integrated balloon catheter delivering therapeutics into the submucosal tissue of a lesion. ii) Photograph of the MNIBC device. iii) Optical microscopy (scale bar = 1 mm) and SEM images (scale bar = 200 µm) highlighting the microneedle electrode design with core‐shell architecture. Reprinted with permission from ref. [107] 2023 Wiley‐VCH GmbH.

Functional tools that can adapt to the environment are key in minimally invasive surgeries. In a recent advance, Lussi et al.^[^ ^72^ ^]^ developed a submillimeter continuous variable stiffness catheter equipped with a phase‐change alloy for precise compliance control. The low‐melting‐point phase‐change alloy incorporated in the catheter quickly changed the stiffness by varying its phase boundary using a controlled radial temperature gradient. This capability enables the catheter to navigate safely in its flexible state and become rigid when needed for surgical operations. As shown in **Figure** **3**A, this novel design adjusted the catheter stiffness dynamically by a factor of 400 (≈20 mN to 8 N) to meet the varying demands of minimally invasive robotic epiretinal membrane peeling surgery. Equipped with a custom microgripper and an electromagnetic navigation system, the catheter enabled manual and precise control of the surgery through a haptic input device that translated the surgeon's hand movements into magnetic field adjustments and catheter movements.

Gopesh et al.^[^ ^73^ ^]^ introduced a hydraulically actuated soft robotic steerable tip microcatheter designed for neuro‐endovascular procedures, particularly for the treatment of cerebral aneurysms. Traditional catheters lack the steerability required to navigate the tortuous vasculature of the brain, often necessitating a trial‐and‐error approach that can increase procedure time and complication rates. The novel microcatheter described in the study features a soft polymer tip with four hydraulic channels for precise, real‐time steering, reducing the reliance on fixed orientation tips and enabling safer, quicker navigation to target sites. Fabricated using advanced microfabrication techniques and engineered hyperelastic materials, the catheter was tested in both ex vivo silicone models and in vivo porcine models, demonstrating successful coil deployment in challenging anatomical locations. This technology shows promise in enhancing the effectiveness of endovascular procedures and non‐vascular procedures requiring fine manipulations in small, complex anatomical locations.

Nguyen et al.^[^ ^74^ ^]^ developed a Miniaturized Soft Robotic Catheter (MSRC) to address the limitations of traditional rigid catheter designs by incorporating a soft, flexible manipulator. The MSRC utilizes soft hydraulic filament artificial muscles for precise navigation and bending, offering enhanced maneuverability in complex anatomical environments. To further improve adaptability, the catheter includes a variable stiffness stabilizing mechanism (VSSM) that allows it to conform to vessels of varying sizes. A highly sensitive pressure‐based sensor was also integrated at the tip to detect contact force between the catheter and surrounding tissue. These combined features enable the MSRC to maintain flexibility and stability, which are imperative for navigating the dynamic cardiothoracic environment. The system was validated through ex vivo cardiac ablation in a simulated right atrium, demonstrating its ability to perform precise maneuvers and sustain structural control under real‐time physiological conditions. The study highlights the potential of soft robotic catheter systems to enhance the safety and efficacy of cardiac ablation and other vascular interventions. Their integration into catheter design marks a significant advancement toward smarter, more responsive medical devices that can reduce operation times and improve surgical outcomes.

In a novel approach towards mitigating heat and improving steering of Magnetic Resonance Imaging (MRI)‐driven microcatheters, Phelan III et al.^[^ ^75^ ^]^ introduced a smart interventional catheter utilizing a quad‐configuration micro coil design that enables significant miniaturization of the catheter, reducing its diameter to 1 mm while maintaining effective electromagnetic steering through Lorentz forces. As shown in Figure 3B, the design addresses the challenge of heat generation by optimizing the power distribution among the coils, mitigating the risk of tissue damage without the need for active cooling mechanisms. The study validated the catheter's performance through MRI‐guided experiments and demonstrated precise steering capabilities in neurovascular and kidney models. The results indicate that the MRI‐driven microcatheter can navigate confined spaces safely and efficiently, making it a promising tool for minimally invasive surgeries.

Repairing luminal defects (e.g., aneurysms, fistulas, vessel perforations) typically requires invasive surgeries that pose risks of complications such as inflammation, perforation, and tissue damage due to sutures or metallic tethers. Toward addressing such risks, Singh et al.^[^ ^76^ ^]^ developed a minimally invasive catheter‐based system with an electroceutical patch to deliver a biocompatible, voltage‐activated adhesive capable of forming strong, watertight seals in dynamic, wet environments without causing additional tissue damage. The catheter has four lumens and retractable nitinol electrodes for precise deployment and activation of the adhesive for site‐specific adhesion on wet tissues. The catheter‐administered adhesive can seal tissue defects up to 2 mm in diameter and withstand over 20000 physiological stress/strain cycles. In addition, the researchers compared the adhesive's adhesion and burst pressure to commercial sealants such as DuraSeal.^[^ ^77^ ^]^ Its functionality was verified in ex vivo and in vivo models, including defect repair in porcine aortas and renal arteries. The electrical stimulation showed minimal electrical interference with surrounding tissues and had low thrombogenic potential, confirmed via in vivo studies on rat hearts.

Magnetic navigation systems have been explored to address the challenges associated with maintaining consistent contact between the catheter tip and the cardiac tissue during ablation procedures, as the external magnetic fields can be used to precisely control the movement and positioning of the catheter tip within the complex cardiac anatomy. A study by Piskarev et al.^[^ ^71^ ^]^ introduced a novel magnetic catheter using a variable stiffness thread (VST) made of a conductive shape memory polymer, which serves as a heater, temperature sensor, and variable stiffness substrate while maintaining biocompatibility. A scalable dipping technique enables precise control over VST thickness (70 µm steps) and electrical resistance. Shown in Figure 3C, the catheter achieves selective bending under a 20 mT magnetic field, with a stiffness change factor of 21. In its rigid state, it can withstand up to 80 mT due to the 21‐fold increase in stiffness. Beyond cardiac ablation, variable stiffness catheters have potential in surgeries involving the stomach, lungs, and brain. The VST fabrication process is adaptable, though further biocompatibility testing is required. Design improvements, including active cooling, alternative insulating materials, and automation, could enhance performance. Additionally, VSTs could be integrated into smart fabrics or aerospace systems requiring adaptable stiffness. Building upon this design, a follow‐up study by the same group^[^ ^78^ ^]^ introduced a fast‐response version of the catheter with enhanced stiffness modulation speed (<2 s) and multiple independently controlled VST segments for complex 3D shaping.

Similarly, Pancaldi et al.^[^ ^79^ ^]^ used magnetic actuation for dynamic steering in the flow‐driven robotic navigation of endovascular microscopic probes through tortuous vasculature. The system relied on viscous stresses and pressure for propulsion throughout the vasculature and homogenous magnetic fields for steering, in combination with nullifying the need for pushing and allowing for the transport of the microprobes at close to the flow speed. Microprobes were fabricated with an outer diameter as small as 120 µm, and probes integrated with magnetic structures as small as 75 µm were reliably tracked with fluoroscopes found in standard operating rooms. Successful demonstration of the microprobes, alongside microcatheters, was shown without observed friction or perfusion in the vessels of ex vivo rabbit ears.

A subset of surgical catheters, ablation catheters, has become widely accepted, as catheter‐based ablation has become an effective treatment for various cardiac arrhythmias, offering a minimally invasive alternative to traditional surgical interventions.^[^ ^80^ ^]^ In particular, the treatment of atrial fibrillation (AF) has seen significant advances, with catheter ablation emerging as a primary management strategy for symptomatic patients.^[^ ^81^ ^]^ Conventional catheter‐based ablation therapy has relied on thermal ablation, but damage to the tissues adjacent to the targeted myocardium has shown tissue‐indiscriminate effects of thermal ablation and presented multiple complications.^[^ ^82^ ^]^ Pulsatile field ablation (PFA) has arisen as a safer alternative to thermal ablation. Xu et al.^[^ ^83^ ^]^ presented a catheter‐integrated fractal microelectrode capable of low‐voltage PFA and minimally invasive interventional sensing, including electrocardiogram (ECG) recording and impedance‐based electrode‐tissue contact detection. The microelectrode system showed irreversible electroporation of cardiomyocytes and subsequent apoptosis due to PFA in vivo, and preservation of the lesion site arteriolar endothelium and nerve fascicles demonstrated myocardial specificity with PFA.

Shen et al.^[^ ^84^ ^]^ developed a bioinspired balloon catheter integrated with stretchable electrodes capable of delivering high‐voltage PFA with uniform electric field distribution. Drawing design inspiration from deep‐sea flounder skeletons and citrus peel geometry, the ultrathin electrodes sustain up to 87% compression without compromising performance. A water lily–inspired transfer printing strategy was employed for one‐step integration of multiple electrodes onto the balloon surface, ensuring accurate spatial placement on the curved geometry. In vivo studies in rabbit and swine models demonstrated consistent electrophysiological isolation and uniform lesion formation.

The integration of force‐sensing capabilities into ablation catheters enables real‐time monitoring of the contact force between the catheter tip and the cardiac tissue, allowing the operator to optimize the delivery of ablative energy and minimize the risk of complications.^[^ ^83^ ^]^ To further improve the safety and efficacy of catheter‐based ablation, the researchers have also explored the use of magnetic navigation systems.^[^ ^71^, ^79^ ^]^ These systems use externally generated magnetic fields to steer a permanent magnet embedded in the catheter tip, eliminating the need for complex internal actuators. This approach can simplify catheter design and potentially reduce complications associated with traditional steerable catheters. Moreover, the integration of magnetic actuation with continuum medical devices offers enhanced dexterity and improved scalability compared to conventional tethered systems.^[^ ^85^ ^]^

Another promising technology in the field of smart catheters is the balloon‐based catheter design, which aims to overcome the limitations of the traditional point‐by‐point RF ablation technique. These balloon‐based catheters, such as the cryoballoon and laser balloon designs, represent a significant departure from the conventional approach, as balloon‐based catheters have the ability to create larger and more uniform lesions in a shorter timeframe, potentially improving procedural efficiency and patient outcomes by reducing the duration of the procedure and limiting the need for multiple, sequential point‐by‐point ablations.^[^ ^86^ ^]^

Catheter ablation of cardiac arrhythmias continues to evolve, with ongoing innovations aimed at improving outcomes and simplifying procedures. The development of smart catheter technologies, such as those with integrated sensors, represents a promising direction, offering the potential to enhance procedural safety, efficacy, and efficiency. One such innovation is the 3D catheter distal force‐sensing technology based on FBG sensors. The technology allows for real‐time monitoring of the contact force between the catheter tip and the cardiac tissue, enabling the operator to optimize the delivery of ablative energy and minimize the risk of complications such as cardiac perforation. This is particularly important as excessive contact force has been identified as a significant contributing factor to ablation‐related complications.^[^ ^87^ ^]^

The emergence of smart catheters that integrate sensing, navigation, and actuation technologies offers continuing improvement in cardiac electrophysiology. Innovations such as shape memory alloy actuators,^[^ ^88^ ^]^ fiber‐optic force sensors,^[^ ^40^ ^]^ and magnetic navigation systems^[^ ^89^ ^]^ collectively enhance the safety, precision, and efficiency of catheter‐based ablation procedures. By improving the ability to access and treat complex cardiac regions, these technologies offer significant potential to reduce procedural complexity and improve patient outcomes.

### 3.2 Therapeutic Catheters

Therapeutic catheters are highly specialized medical devices meticulously engineered to precisely administer therapeutic agents to targeted anatomical locations within the body. These catheters play a pivotal role in a wide array of medical disciplines, including cardiology and oncology, where they are utilized to administer treatments such as targeted drug delivery precisely. The catheters employ intricate microstructures in their design to substantially augment the device's flexibility and maneuverability for various applications, thereby minimizing the incidence of systemic adverse effects seen during these procedures. The significance of therapeutic delivery catheters emanates from their capacity to provide minimally invasive treatment modalities, thereby contributing to improved patient outcomes and potentially reducing recovery times compared to traditional surgical interventions.

One of the most critical areas for therapeutic intervention is cancer treatment, particularly in preventing metastasis. Circulating tumor cells (CTCs), which detach from primary or metastatic tumors and enter the bloodstream, play a central role in cancer metastasis.^[^ ^90^ ^]^ To address this, Wang et al.^[^ ^91^ ^]^ developed a flexible electronic catheter capable of capturing and eliminating CTCs in vivo. The catheter integrates electrospun nanofibers functionalized with epithelial cell adhesion molecule antibodies for specific CTC capture and liquid metal‐polymer composite electrodes for cell ablation. This dual‐function design enhances biorecognition and localized tumor cell destruction without harming surrounding tissues. Validated in a rabbit model, the system achieved a CTC killing efficiency of 100% with minimal off‐target effects or systemic toxicity. This therapeutic catheter offers a minimally invasive strategy for reducing metastatic risk by clearing CTCs directly from the bloodstream.

Rivkin et al.^[^ ^11^ ^]^ introduced a microcatheter, the integrated self‐assembled catheter (ISAC), that directly incorporates sensing and actuation functionalities into the catheter wall using photolithographically processed, self‐assembling polymer films. With a diameter as small as 0.1 mm, this catheter overcomes the limitations of conventional designs that rely on manually assembling components. Wafer‐scale fabrication and 3D self‐assembly techniques enable high integration density and mechanical robustness. Key functionalities include micromanipulation via conductive polymer actuators, targeted fluid delivery, and high‐resolution magnetic navigation through embedded anisotropic magnetoresistance sensors. As shown in Figure 3D, the catheter's fluidic delivery performance was validated using a model channel mimicking an aneurysm, while ex vivo testing on mouse esophageal and gastric tissues demonstrated adaptability to complex physiological conditions. Additionally, the device features four flexible digits capable of grasping and manipulating microscopic structures, enabling applications such as biopsies, implant placement, and neurovascular interventions.

Kim et al.^[^ ^92^ ^]^ demonstrated a submillimeter soft continuum robot with omnidirectional navigation capabilities upon magnetic actuation. The soft polymer matrix containing embedded ferromagnetic particles could be navigated through narrow and tortuous vasculature, and the core of the robot body was composed of a nitinol core for mechanical support and trackability. To further increase the capability of advancing through tortuous anatomy, a hydrogel skin was grown onto the elastomer chains of the robot's soft polymer surface, reducing surface friction by a factor of ten. Though validated in vitro, further preclinical studies exploring the biocompatibility of the device, particularly with respect to the embedded ferromagnetic particles, are needed before further steps toward clinical translation.

These magnetic catheter robots show the promise of new technologies that offer flexibility and controllability to improve minimally invasive surgeries. However, most of these technologies are single‐function and lack triaxial force sensing, which limits their clinical applications. Fu et al.^[^ ^93^ ^]^ proposed a multifunctional magnetic catheter robot with magnetic actuation steering and triaxial‐force sensing capabilities for high‐sensitivity 3D force measurements. The 1‐mm‐diameter FBG used to sense triaxial force relies on only one optical fiber, effectively miniaturizing the sensor and improving its integration into the magnetic catheter by minimizing assembly errors associated with larger numbers of optical fibers. The three‐channel catheter—composed of a silicone composite to maintain flexibility—contains a working channel, a magnetic channel with multiple magnetic segments inserted, and a sensor channel where the force sensor is integrated. The system was validated in vitro in lung and stomach phantoms and ex vivo in a porcine kidney via palpation of simulated nodules, polyps, and tumor lumps, respectively, in each of the investigated tissue types.

To address poor surgical outcomes in intraventricular hemorrhage treatment due to occlusion of existing intraventricular devices, Yang et al.^[^ ^94^ ^]^ introduced a novel self‐clearing implantable catheter enabled by magnetic micro‐actuators. The magnetic micro‐actuators were fabricated in two shapes, a simple rectangular cantilever and a serpentine flexure, and controlled externally via time‐varying magnetic fields. The serpentine flexure showed an increased ability to break down obstructive macroscopic hematoma over previous micro‐actuators. The system demonstrated statistically significant delays in occlusion due to hematoma and reduction in the size of the hematoma in a continuous flow environment in vitro, and these results were verified in vivo in a porcine model. A further iteration of the system to include a similar number of drainage pores to existing intraventricular devices, improve accuracy in device placement, and reduce the risk of infection is needed for further validation of the treatment.

In‐vivo bioprinting has appeared as a method for the direct fabrication of artificial tissues and medical devices on living tissues, but existing methods are limited in their applications due to the necessity of open surgery for printing on internal organs. In response, Zhou et al.^[^ ^95^ ^]^ developed a ferromagnetic soft catheter (FSCR) for minimally invasive in‐vivo bioprinting, as shown in Figure 3E. The soft polymer body of the FSCR system is embedded with hard magnetic ferromagnetic particles, which are controlled by the magnetic field imposed by four numerically controlled motor‐driven permanent magnets. Printing of multiple patterns on planar surfaces was demonstrated, and a functional hydrogel was printed on a porcine tissue phantom and in vivo on a rat liver. Thus, a proof‐of‐concept FSCR for minimally invasive tissue engineering was demonstrated.

To address the persistent challenge of CAUTIs, recent smart catheter designs have embraced innovative surface engineering and multifunctional material strategies. Won et al.^[^ ^96^ ^]^ developed a dual‐layer nanoengineered urinary catheter featuring an inner silver nanoparticle layer for immediate bactericidal action and an outer porous zinc coating to enable sustained, zero‐order silver ion release, collectively achieving >99.9% bacterial reduction and suppressed biofilm formation over long‐term implantation. Puertas‐Segura et al.^[^ ^97^ ^]^ introduced an antibiotic‐free composite coating using silver‐lignin nanoparticles and zwitterionic polymers, assembled via laccase‐mediated enzymatic grafting, which offered bactericidal effects under dynamic flow without signs of cytotoxicity. In a complementary approach, Zhou et al.^[^ ^98^ ^]^ proposed a fundamentally different strategy, an artificial intelligence (AI)‐optimized geometric design that disrupts bacterial upstream swimming through asymmetric obstacle patterns within the catheter lumen, reducing bacterial colonization without relying on biochemical agents. These smart catheters employ different strategies, all aimed at enhancing therapeutic performance through infection prevention.

### 3.3 Imaging Catheters

Imaging catheters are a rapidly evolving class of smart catheter technologies that enable real‐time visualization of internal anatomy, providing critical feedback for diagnostic, interventional, and surgical procedures.^[^ ^18^ ^]^ Unlike traditional imaging tools that rely on external visualization, imaging catheters integrate advanced modalities such as optical coherence tomography (OCT), intravascular ultrasound (IVUS), and MRI, among others. With imaging catheters, clinicians can visualize tissue morphology, device placement, and procedure outcomes with greater precision without the need for contrast agents or ionizing radiation.

MRI‐compatible imaging catheters are also increasing in demand for procedures requiring soft tissue contrast and radiation‐free imaging. Heidt et al.^[^ ^99^ ^]^ custom‐designed catheters with an active receive tip‐coil to improve visibility and navigation capabilities. The feasibility of the system was demonstrated in a porcine model, using real‐time MRI‐compatible catheters to perform coronary interventions without radiation or contrast media. A recent study further explored this approach by guiding balloon dilation and stenting entirely under MRI in a swine model, using a commercially available scanner and custom‐engineered catheters.^[^ ^100^ ^]^

Kellnberger et al.^[^ ^101^ ^]^ introduced a hybrid imaging technique combining near‐infrared fluorescence (NIRF) and IVUS, shown in Figure 3F, enhanced with indocyanine green dye to assess atherosclerotic plaques in excised human coronary arteries. Greater dye uptake was observed in advanced fibroatheromas, while lower uptake was seen in early or calcified lesions. A strong correlation (i.e., r = 0.668) between the fluorescence signal and CD68‐positive macrophage density confirmed the technique's sensitivity to inflammation. This dual‐modality approach enables effective visualization of plaque composition and may improve risk assessment in coronary artery disease.

Newer catheter systems are also borrowing inspiration from biology and physics to develop novel imaging techniques. Sutton et al.^[^ ^102^ ^]^ developed a biologically inspired catheter that uses electrical impedance mapping, like the electrolocation ability of electric fish, to navigate blood vessels in real‐time without external imaging or radiation. Mimicking the electrolocation ability mechanism, this smart catheter emits a weak electric field through tip‐mounted ring electrodes and records changes in impedance caused by variations in local vessel geometry. The system maps these impedance signals to a preoperative 3D vascular model to localize the catheter within the vessel tree in real‐time without the need for external tracking or image registration. This approach not only enhances safety and precision during endovascular procedures but also provides a cost‐effective and scalable alternative. The study presented a new non‐fluoroscopic navigation technique to supplement existing imaging methods.

Cao et al.^[^ ^103^ ^]^ introduced a smart intravascular photoacoustic imaging catheter that overcomes key limitations of conventional designs through a novel collinear configuration. Unlike non‐collinear systems that suffer from limited overlap between optical and acoustic fields, this catheter aligns both waves along the same axis within a compact 1.6 mm housing, enabling consistent signal generation over a depth exceeding 6 mm. This innovation allows chemically specific imaging of lipid‐rich plaques with a spatial resolution of 80 µm axially and about 400 µm laterally. As shown in Figure 3G, the catheter integrates a side‐viewing, 45°‐polished multimode fiber, and a precisely positioned ultrasound transducer, enabling real‐time co‐registered intravascular photoacoustic imaging and IVUS imaging.

Adding to these imaging technologies, fiber‐optic sensor integration is playing a growing role in catheter innovation. These sensors offer high sensitivity, compact design, and real‐time tracking, which are vital for applications like catheter navigation and physiological monitoring. Ha et al.^[^ ^104^ ^]^ developed a novel method for improving catheter tracking in endovascular interventions, combining electromagnetic tracking, FBGs for shape sensing, and fluoroscopy. The approach minimizes fluoroscopic exposure by using fiber sensors for 3D shape reconstruction and electromagnetic tracking for localization, with twist compensation via fluoroscopic images. This method significantly enhances accuracy, achieving low root‐mean‐square errors in 3D experiments.

As imaging resolution, catheter flexibility, and sensor integration continue to evolve, imaging catheters are redefining how clinicians approach diagnosis and intervention. The ability to offer both access and visualization—especially in delicate or anatomically complex areas—makes imaging catheters indispensable tools in the future of minimally invasive and precision medicine.

### 3.4 Drug‐Eluting Catheters

Drug‐eluting smart catheters represent a significant advance in catheter technology, combining the benefits of localized drug delivery with advanced features such as sensors, actuators, and magnetic navigation. These catheters offer the potential for improved treatment outcomes and reduced complications in various medical procedures.

To address the limitations of traditional drug‐eluting balloon (DEB) systems, Lee et al.^[^ ^105^ ^]^ developed a microneedle drug‐eluting balloon (MNDEB) for enhanced drug delivery to vascular tissues. As shown in Figure 3H, MNDEB incorporates microneedles (MNs) onto the surface of a DEB using a conformal transfer molding process, which improves mechanical adhesion and prevents detachment during deployment. By directly penetrating the vascular tissue, the MNDEBs facilitate deeper and more efficient drug delivery compared to standard DEBs, which rely on passive diffusion. In vivo testing in a rabbit model demonstrated superior drug delivery efficiency of the MNDEB compared to conventional DEBs, with significantly higher drug concentrations delivered to the targeted site. Additionally, the safety of the MNDEB was confirmed, showing no significant damage to vascular integrity. These findings highlight the potential of MNDEBs to improve therapeutic outcomes in treating vascular diseases such as atherosclerosis and in‐stent restenosis by addressing the limitations of conventional DEB technologies.

Huang et al.^[^ ^106^ ^]^ demonstrated another DEB technology, integrating tip‐separable MNs on the surface of a drug‐loaded balloon for arteriosclerosis treatment. A ring‐laser fiber introduced into the catheter's inner shaft melts the phase‐change material in the MN tips upon emitting a near‐infrared laser to decrease the shear force required to detach the tips from the bases as they become embedded in the tissue. The tip‐separable microneedle design was key in maintaining the strength necessary to penetrate the blood vessel, then allowing for MN detachment for effective drug delivery. Significant increases in drug delivery efficiency as compared to the standard DEB were demonstrated in vivo in atherosclerotic rabbit models.

A separate group, Huang et al.,^[^ ^107^ ^]^ designed a catheter including multimodal core‐shell microneedles, where each MN tip possessed an individual functionality, totaling eleven functions. In combining real‐time biochemical sensing, myoelectric sensing, electroporation, and drug delivery, the system shown in Figure 3I, was successfully demonstrated ex vivo on a porcine bladder and in vivo on anesthetized rabbits for precise localization and ablation of lesions and drug delivery into the submucosal tissue region surrounding the MNs. Though the simulated clinical scenario focused on endoscopy‐guided surgery for the diagnosis and treatment of bladder diseases, the system was designed for minimally invasive tissue penetration in various surgical applications.

## 4 Key Technologies in Smart Catheter

### 4.1 Sensor Integration and Fabrication Technologies

The fabrication of smart catheters increasingly relies on a hybrid strategy that brings together traditional microfabrication methods with emerging soft electronics, self‐assembly techniques, and advanced integration approaches designed specifically for flexible, curved surfaces.^[^ ^108^, ^109^, ^110^ ^]^ Established processes like photolithography, soft lithography, and thin‐film deposition, originally optimized for flat semiconductor wafers, are now being adapted to meet the unique geometric and mechanical requirements of catheter‐based devices. While these methods offer the precision needed to embed microscale sensors and circuits, the main challenge lies in integrating these components conformally onto flexible, stretchable catheter surfaces without compromising mechanical compliance, biocompatibility, or functionality.^[^ ^42^ ^]^ As catheter architectures become more complex, integration methods must also account for localized strain, frequent bending, and continuous exposure to physiological fluids. To overcome these obstacles, researchers have turned to innovative fabrication strategies such as stress‐induced self‐rolling, transfer printing, femtosecond laser micromachining, and the use of stretchable or serpentine interconnects.^[^ ^11^, ^108^, ^109^, ^110^, ^111^ ^]^

Traditional MEMS techniques, rooted in semiconductor manufacturing and characterized by a layer‐by‐layer fabrication process, continue to serve as a foundational approach for sensor integration in smart catheters. For instance, Li et al.^[^ ^60^ ^]^ demonstrated a temperature‐sensing catheter in which gold and titanium microelectrodes were patterned on polyimide substrates via photolithography and subsequently encapsulated with Parylene C before being helically wrapped around the catheter shaft. Similar approaches have been widely employed for the development of pressure and force sensors, utilizing either conventional or soft lithography to pattern thin‐film gold electrodes.^[^ ^35^, ^58^, ^60^, ^66^ ^]^ These MEMS‐based methods are well‐suited for achieving high accuracy and batch‐to‐batch consistency; however, conformally integrating these sensors onto the catheter's curved and dynamic surfaces remains a significant challenge.^[^ ^42^ ^]^

**Figure 4:**
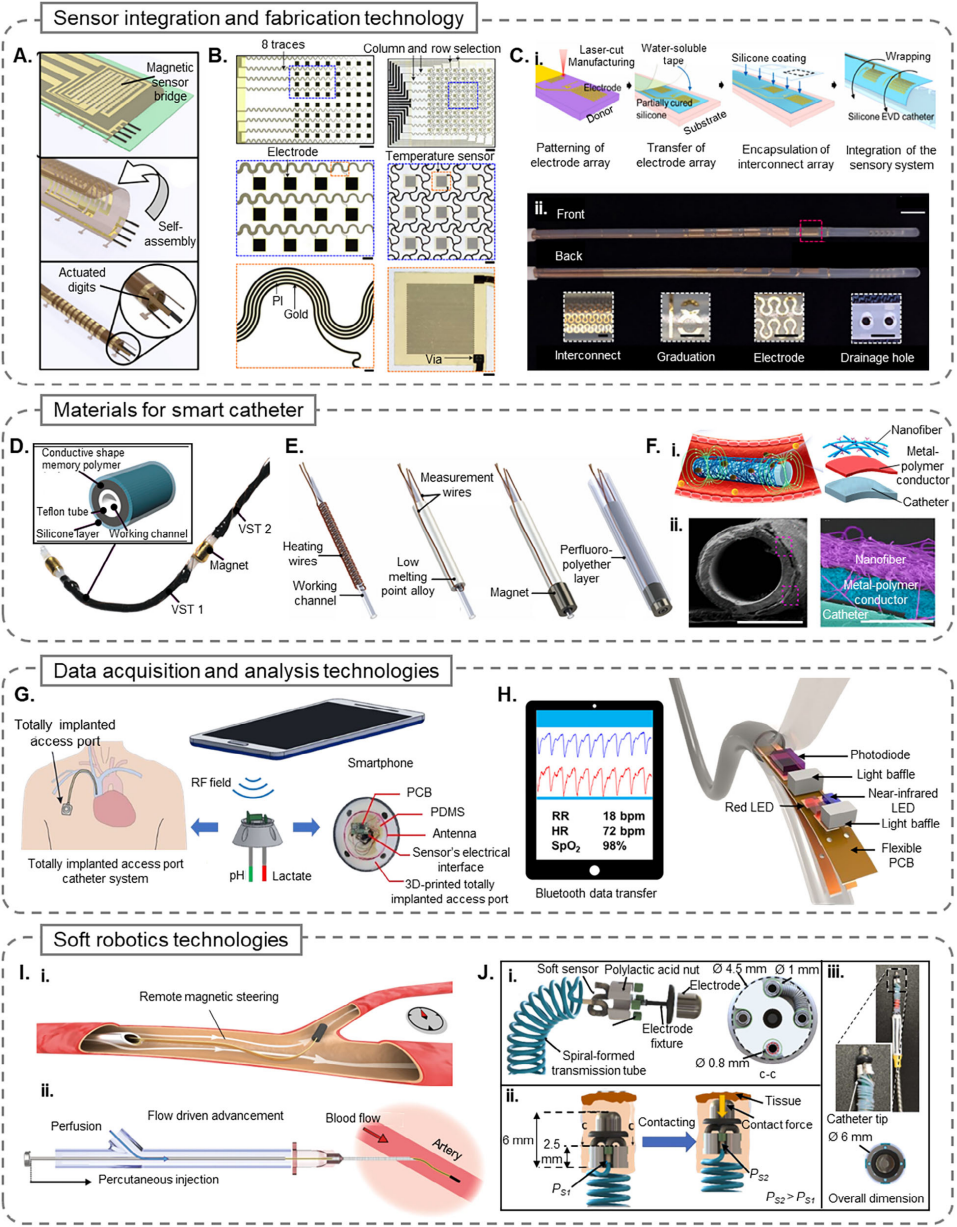
A) Fabrication of ISACs by layering thin planar films followed by self‐rolling into tubular structures. Reprinted with permission from ref. [11] B) Planar arrays of gold‐based electrodes and temperature sensors prior to catheter integration. Multiple photolithography steps were used to create the electrodes and sensors. Reprinted with permission from ref. [19] C i) Key steps in device fabrication, including electrode array patterning, transfer, encapsulation, and integration with the catheter body. ii. Photographs showing the completed smart catheter with magnified views of critical features such as interconnects, graduation marks, electrodes, and drainage holes (scale bar = 1 mm). Reprinted with permission from ref. [64] 2024 Elsevier B.V. D) Structure and working principle of a variable stiffness thread, consisting of a polytetrafluoroethylene tube with an internal conductive shape memory polymer layer and external silicone encapsulation, enabling tunable stiffness for smart catheter applications. Reprinted with permission from ref. [71] 2022 The Authors. Advanced Functional Materials published by Wiley‐VCH GmbH. E) Manufacturing process of a continuous variable stiffness catheter, involving the coiling of heating wires, coating with a low melting point alloy, attachment of a permanent magnet, and encapsulation with a 150 µm perfluoropolyether layer for mechanical stability. Reprinted with permission from ref. [72] 2021 The Authors. Advanced Science published by Wiley‐VCH GmbH. F i) Schematic showing a flexible electronic catheter designed for in vivo capture and elimination of circulating tumor cells. ii) SEM images highlighting the surface morphology of the metal‐polymer conductor/nanofiber‐modified catheter (scale bars: 1 cm and 50 µm). Reprinted with permission from ref. [91] 2022, American Chemical Society. G) Wireless data acquisition from implantable sensors using a totally implantable access port, RF transmission, and smartphone‐based real‐time monitoring. Reprinted with permission from ref. [23] 2022 The Authors. Published by Elsevier Ltd. H) Enlarged view of a miniaturized flexible PCB‐based optical sensor probe, incorporating light sources, detectors, and blocking layers, fully encapsulated in biocompatible silicone for continuous physiological monitoring. Reprinted with permission from ref. [21] Copyright 2021, The American Association for the Advancement of Science. I i) Ultra‐flexible µ‐probes guided by external magnetic fields for targeted navigation into small vessels. ii. Strategies for stabilizing probe deployment during injection, including perfusion‐assisted tensioning to maintain structural integrity during vessel entry. Reprinted with permission from ref. [79]. J i) Design layout of a soft robotic catheter tip integrating an array of stretchable sensors. ii. Demonstration of mechanical adaptation between free and tissue‐contacted states. iii. Overview of the full robotic catheter prototype illustrating dimensions and assembly features. Reprinted with permission from ref. [74] 2023 Elsevier B.V.

To overcome such geometric and mechanical constraints, self‐assembly methods have gained increasing interest. A prominent example is the Integrated Sensor Array Catheter (ISAC) developed by Rivkin et al.,^[^ ^11^ ^]^ where a planar multilayer stack comprising polyimide, conductive traces, and magnetic components undergoes stress‐induced self‐rolling, forming a compact tubular structure. As shown in **Figure** **4**A, this transformation from a 2D layout into a 3D “Swiss‐roll” geometry facilitates high‐density sensor integration while significantly reducing the need for manual assembly.

Transfer printing represents another widely adopted strategy for sensor integration, particularly for assembling prefabricated microstructures onto elastomeric or balloon‐type catheter substrates.^[^ ^64^, ^65^, ^66^ ^]^ Han et al.^[^ ^19^ ^]^ utilized this technique to fabricate a stretchable, multiplexed sensor array capable of spatiotemporal physiological mapping. The device, shown in Figure 4B, consists of multiple functional layers—including gold electrodes, temperature sensors, and 3D buckled pressure sensors—initially patterned on silicon wafers and subsequently transferred onto catheter balloons. To preserve mechanical compliance during inflation and deflation cycles, serpentine interconnects were incorporated to enhance stretchability without compromising electrical continuity.

Several modifications of transfer printing have been developed to accommodate more complex geometries and sensor types. Shen et al.,^[^ ^84^ ^]^ for example, employed a one‐step transfer technique using a custom‐built apparatus inspired by the water‐lily structure to bond high‐voltage PFA electrodes onto an inflatable balloon catheter. In another study, Ji et al.^[^ ^65^ ^]^ utilized a water‐floatation transfer method to laminate ultrathin organic transistor‐based sensors directly onto ventricular catheters.

Direct patterning techniques also offer viable solutions for integrating functional elements onto nonplanar catheter surfaces. Femtosecond laser micromachining enables precise and mask‐free fabrication on curved substrates. Shang et al.^[^ ^36^ ^]^ utilized this method to engrave microchannels along a polyurethane catheter's outer surface, subsequently coating the channels with a piezoresistive material to enable circumferential pressure sensing with full 360° spatial resolution. Similarly, Yi et al.^[^ ^64^ ^]^ employed a combination of femtosecond laser ablation, tape‐assisted transfer, and plasma bonding to embed electrode arrays into PDMS membranes, which were then mounted onto silicone catheters for neurosurgical applications. The detailed integration process is illustrated in Figure 4C.

From these examples, it is clear that a growing trend is emerging toward hybrid fabrication and integration strategies for the development of smart catheters. Whether through traditional lithography or advanced techniques such as stress‐induced self‐rolling, femtosecond laser micromachining, or multilayer transfer printing, these methods are collectively enabling the realization of catheters that are miniature, mechanically compliant, and capable of conformal integration with soft, curved anatomical environments. To make trade‐offs explicit, we provide a comparison of major integration technologies in Table S2 (Supporting Information), focusing on fabrication precision, repeatability, robustness, and challenges faced due to curved surfaces, alignment and adhesion. Typical applications in smart catheters are also discussed in the table.

### 4.2 Materials for Smart Catheters

Traditional catheters struggle with flexibility and control, often requiring multiple instruments to perform a single procedure. In modern smart catheter technology, the choice of materials plays a crucial role in overcoming limitations such as flexibility, control, and sensor integration.^[^ ^28^ ^]^

For example, phase‐changing materials are used to enable variable stiffness in catheters. Piskarev et al.^[^ ^71^ ^]^ developed a magnetic catheter, shown in Figure 4D, using conductive shape‐memory polymers, offering both flexibility and improved temperature sensitivity for enhanced control in cardiac surgeries. Similarly, Lussi et al.^[^ ^72^ ^]^ demonstrated a submillimeter catheter that uses low‐melting point alloys to switch between flexible and rigid states, as shown in Figure 4E. This allows the catheter to navigate through complex anatomical structures and apply needed forces during procedures. Nguyen et al.^[^ ^74^ ^]^ developed a soft robotic catheter with a variable stiffness stabilizing mechanism made from phase‐changing materials, which maintains shape and dexterity during navigation, enabling more precise operations during vascular surgery.

Nanomaterials like graphene and carbon nanotubes address conductivity and bacterial adhesion challenges. The flexible electronic catheter developed by Wang et al.^[^ ^91^ ^]^ achieves its smart therapeutic functionality primarily through strategic material modification. As shown in Figure 4F, the catheter is coated with an electrospun nanofiber membrane that provides a high surface area and is functionalized with epithelial cell adhesion molecule antibodies to enable selective capture of circulating tumor cells. To add therapeutic capability, the researchers incorporated a liquid metal‐polymer conductor layer composed of gallium‐indium alloy and polyvinyl alcohol, forming highly stretchable and conductive electrodes. This material combination allows the catheter to perform irreversible electroporation, selectively ablating adhered tumor cells without damaging nearby healthy tissue. Similarly, in a study by Baburova et al.,^[^ ^112^ ^]^ carbon nanowires are embedded in a soft magnetic robot to enhance mechanical strength and magnetic responsiveness. This improves the robots' ability to remove biofilms from catheter surfaces under external magnetic control efficiently.

In addition to advanced bulk materials, surface coatings play a pivotal role in enhancing the functionality and biocompatibility of smart catheters, particularly in addressing infection risk and tissue compatibility.^[^ ^113^, ^114^ ^]^ In a study by Puertas‐Segura et al.,^[^ ^97^ ^]^ an enzymatically built nano‐enabled coating was fabricated using silver‐phenolated lignin nanoparticles combined with carboxybetaine‐based zwitterionic polymers on a urinary catheter. The resulting surface exhibited potent bactericidal and antifouling properties under dynamic flow conditions, while maintaining excellent in vivo biocompatibility. Similarly, Won et al.^[^ ^96^ ^]^ developed a dual‐layer antimicrobial coating for urinary catheters, combining a zwitterionic poly(carboxybetaine methacrylate) base layer to resist biofilm formation with a silver nanoparticle top layer for immediate antibacterial action. Surface coatings on catheters can also be used as sensors as shown in a study by Li et al.^[^ ^53^ ^]^ A temperature‐sensing hydrogel coating was applied to the surface of catheters to enable real‐time thermal monitoring during clinical use. The hydrogel matrix supports flexible integration of thermoresponsive materials, while maintaining stretchability, optical transparency, and minimal cytotoxicity. Material choices for smart catheters are compared in Table S3 (Supporting Information), highlighting the benefits of specific materials for navigation, sensing and therapy versus risks such as heating, fatigue, encapsulation leakage, and long‐term biocompatibility.

### 4.3 Data Acquisition and Analysis Technologies

Traditional catheter systems relied heavily on external data recording and manual monitoring, often leading to delays in diagnosis and treatment adjustments. Technologies like Bluetooth and NFC overcome this by enabling long or short‐range wireless communication between the catheter and external devices. This communication provides for immediate data retrieval and analysis, improving the speed and accuracy of diagnostic and therapeutic procedures.

Gil et al.^[^ ^23^ ^]^ introduced NFC microelectronics into an implanted access port catheter to enable wireless interrogation of pH and lactate biosensors, as shown in Figure 4G. This system allows healthcare providers or patients to retrieve biosensor data using a smartphone equipped with an NFC interface. The NFC functionality provides both data acquisition and energy harvesting, eliminating the need for batteries and enabling continuous health monitoring in a noninvasive and patient‐friendly way.

A key advance in the wireless catheter‐type oximeter developed by Lu et al.^[^ ^21^ ^]^ lies in its real‐time data acquisition framework, which transforms conventional passive catheters into intelligent, interactive monitoring platforms. Unlike fiber‐optic oximeters that rely on rigid glass waveguides and bedside hardware, the system shown in Figure 4H incorporates a soft, miniaturized optoelectronic probe connected to a Bluetooth‐enabled control module, enabling continuous wireless transmission of physiological data. An onboard microcontroller unit handles real‐time signal processing and wireless transmission, which samples, filters, and transmits the data to a custom graphical user interface for visualization and control. This closed‐loop wireless setup enables high‐resolution heart rate, respiratory rate, and localized oxygen saturation monitoring. Wireless options and on‐device processing for catheter systems are compared in Table S4 (Supporting Information), focusing on range, power budget/harvesting, bandwidth, latency, and clinical workflow implications.

### 4.4 Soft Robotics and Electromagnetic Actuation

The addition of robotic technologies has transformed traditional catheters from passive tools into intelligent devices with enhanced precision, control, and surgical and therapeutic capabilities. By integrating robotic features such as remote manipulation and automated navigation, these advanced catheters can perform complex procedures with greater accuracy, improving patient safety and treatment outcomes. Robotic catheters often include actuators for precise movement and responsive adjustments during procedures. These innovations have paved the way for minimally invasive, image‐guided surgeries, making treatments faster, less invasive, and more effective.

Robotic technologies are being incorporated into catheters to improve their therapeutic functionalities. For example, Baburova et al.^[^ ^112^ ^]^ developed a magnetic soft robot to address biofilm‐related infections in urethral catheters, a common issue compromising device function and requiring catheter replacement. The soft robot eradicates biofilm through mechanical force by harnessing a rotating magnetic field. This robotic approach provides minimally invasive cleaning, significantly prolonging catheter functionality and reducing infection risks without removal or replacement. Similarly, Yang et al.^[^ ^94^ ^]^ introduced a robotic self‐clearing catheter for managing intraventricular hemorrhage. Equipped with magnetic microactuators, this catheter autonomously clears blood clots, obstructing cerebrospinal fluid flow when activated externally, thereby extending catheter usability and reducing clogs in critical stroke cases. Zhou et al.^[^ ^95^ ^]^ developed a ferromagnetic robotic soft catheter system for in vivo bioprinting, marking a potential breakthrough in minimally invasive tissue engineering. Utilizing magnetic actuation, this robotic catheter enables precise control for 3D bioprinting directly on internal organ surfaces, as demonstrated in animal models. Such robotic catheters can customize material deposition in hard‐to‐reach areas, presenting transformative potential for tissue repair and implant integration.

Smart catheters with robotic features can also be used for precise control and improved navigation, particularly during minimally invasive surgeries. A robotic catheter used for neurovascular interventions has been introduced by Pancaldi et al.,^[^ ^79^ ^]^ who demonstrated a flow‐driven robotic navigation system for ultra‐flexible endovascular probes, integrating robotics into catheter systems to navigate through the brain's complex vasculature. As shown in Figure 4I, the system uses blood flow and magnetic actuation to autonomously guide the catheter through vessel networks, reducing external manipulation. Another robotic soft catheter system developed by Nguyen et al.^[^ ^74^ ^]^ features omnidirectional movement and variable stiffness control for vascular interventions, as shown in Figure 4J. The system uses hydraulic filament artificial muscles for flexible navigation and a robotic stabilizing mechanism to secure the catheter during cardiac procedures. This robotic catheter's stability and force‐sensing capabilities enhance precision in vascular surgeries, particularly under dynamic conditions, making it highly suitable for minimally invasive cardiac treatments. Gopesh et al.^[^ ^73^ ^]^ designed a hydraulically actuated robotic microcatheter to improve navigation and control in endovascular procedures for cerebral aneurysms. This soft robotic catheter achieves precise 3D steering without a guidewire, utilizing hyper‐elastic polymers to enhance maneuverability through complex vasculature. The robotic design allows for accurate coil deployment, demonstrating substantial potential for improving neuro‐interventional treatments. Various catheter actuation and navigation systems are given in Table S5 (Supporting Information), comparing steerability, response speed, and system complexity with typical applications in smart catheters.

### 4.5 Pre‐Clinical Translation of Smart Catheter Technologies

The clinical translation of smart catheter technologies depends on pre‐clinical validation that demonstrates safety, efficacy, and functional reliability in physiologically relevant environments. While a growing number of studies have introduced innovative sensing, therapeutic, and imaging catheters, only subsets have undergone systematic in vivo or ex vivo evaluations. This section summarizes the primary achievements demonstrated in pre‐clinical models, highlighting both their translational promise and remaining limitations.

Several studies advanced from in vitro validation to in vivo testing using animal models to confirm biocompatibility and real‐time monitoring capability. Shang et al.^[^ ^36^ ^]^ demonstrated a flexible pressure‐sensing catheter in a porcine model of obstructive sleep apnea, successfully mapping soft tissue pressure along the upper airway with results that correlated with computerized tomography and polysomnography data. Similarly, Klinker et al.^[^ ^59^ ^]^ validated a stretchable balloon catheter for blood flow monitoring in porcine femoral arteries under physiologically relevant pressures, confirming a stable signal response during balloon inflation and vascular contact. Lu et al.^[^ ^21^ ^]^ developed a soft wireless oximetry catheter tested in rats, which reliably captured cardiac oxygenation and heart rate over eight weeks without tissue irritation. These examples show strong translational potential, as they replicate in vivo dynamic physiological conditions rather than static in vitro setups. These platforms demonstrate biocompatibility and real‐time data validation in large‐animal models. However, chronic implantation and scaling to human anatomical geometries remain ongoing challenges.

Smart therapeutic catheters have achieved significant milestones in targeted drug delivery and disease treatment under in vivo conditions. Wang et al.^[^ ^91^ ^]^ validated a biofunctionalized electronic catheter in a rabbit model, effectively capturing and destroying circulating tumor cells with 100% killing efficiency and no systemic toxicity. Won et al.^[^ ^96^ ^]^ tested dual‐layer antimicrobial urinary catheters with silver nanoparticle and zinc coatings, achieving >99.9% bacterial reduction in long‐term animal studies. Lee et al.^[^ ^105^ ^]^ and Huang et al.^[^ ^106^ ^]^ demonstrated microneedle drug‐eluting balloon catheters in rabbit models of atherosclerosis, confirming superior local drug retention and vascular safety compared to conventional DEB systems. These devices confirm safety and functionality, yet long‐term degradation, systemic biocompatibility, and regulatory biostability data are limited, and manufacturing reproducibility and sterilization validation remain barriers to clinical adoption.

Robotic and magnetically actuated catheters have shown promising ex vivo and in vivo performance in navigation and minimally invasive surgery. Gopesh et al.^[^ ^73^ ^]^ demonstrated a hydraulically actuated microcatheter capable of real‐time steering during in vivo porcine neuro‐endovascular procedures. Nguyen et al.^[^ ^74^ ^]^ validated an MSRC for cardiac ablation in ex vivo heart models, achieving stable contact forces and precise navigation. Phelan et al.^[^ ^75^ ^]^ achieved MRI‐guided microcatheter steering in neurovascular and renal models, demonstrating controlled navigation without excess heat generation. Yang et al.^[^ ^94^ ^]^ verified a magnetically actuated self‐clearing ventricular catheter in a porcine hemorrhage model, which significantly delayed occlusion and reduced clot size, suggesting translational potential for stroke treatment. These catheters were validated for short‐term in vivo use but still require design simplification, chronic safety assessments, and human‐scale validation.

Imaging‐enabled catheters have progressed furthest toward clinical application, due to the maturity of underlying imaging modalities such as IVUS, OCT, and MRI. Heidt et al.^[^ ^99^ ^]^ performed in vivo coronary interventions in pigs using MRI‐compatible catheters with integrated receive coils, successfully guiding stenting procedures without contrast agents. Kellnberger et al.^[^ ^101^ ^]^ conducted ex vivo imaging of human coronary arteries using hybrid IVUS–fluorescence catheters, confirming plaque sensitivity and inflammation correlation. These imaging systems are closest to clinical deployment, as their safety profiles align with established catheter‐based imaging technologies.

## 5 Commercially Available Smart Catheters

The global market for smart catheters incorporating sensors is experiencing strong growth, fueled by rising cases of cardiovascular, urological, and chronic diseases, an aging population, and the increasing adoption of minimally invasive procedures. According to The Business Research Company, the market size is expected to grow from $3.75 billion in 2024 to $5.64 billion by 2029 at a compound annual growth rate of 8.4%.^[^ ^115^ ^]^ Similarly, InsightAce Analytic projects the market to expand from $3.8 billion in 2024 to $8.6 billion by 2034, with a compound annual growth rate of 8.5%, highlighting additional drivers such as technological advances and increased investments from governmental and private sectors.^[^ ^116^ ^]^ Together, these trends position sensor‐based smart catheters as pivotal tools in advancing personalized medicine and improving healthcare delivery. This growth is primarily driven by the rising need for sophisticated diagnostic and therapeutic tools across multiple medical specialties.

Smart catheters integrate innovations in sensor technology, data analytics, and minimally invasive techniques to improve patient outcomes and streamline clinical workflows. Multiple companies have developed specialized smart catheters targeting diverse applications, from cardiovascular diagnostics to chronic pain management. **Table** **3** provides some commercially available devices that highlight the breadth and functionality of the current market.

**Table 3:** List of Some Commercially Available Smart Catheters.

Device Name	Clinical Application	Smart Technology Integration	Technical Specifications & Innovation	Manufacturer	Reference
ThermoCool SmartTouch®	Cardiac ablation	Real‐time contact force feedback allows precise ablation	RF energy delivery, contact force sensor, bi‐directional catheter	Biosense Webster (Johnson & Johnson)	[117]
Map‐iT Catheter	Cardiac electrophysiology (robotic surgery)	Magnetic guidance with integrated feedback for tissue navigation	Electromagnetic sensors, robotic‐assisted catheter steering	Stereotaxis	[118]
TactiCath Catheter	Cardiac ablation	Dual optical sensors for real‐time contact force measurement	Axial and lateral forces measurement with sub‐gram accuracy, integrates with EnSite Precision system	Abbott Laboratories	[119]
TactiFlex Catheter	Cardiac ablation	Contact force sensing with real‐time visual feedback; compatible with EnSite X system	Laser‐cut flexible tip for improved stability and irrigation, visual indicators for tip deflection and contact force	Abbott Laboratories	[121]
FloTrac Hemodynamic Monitoring System	Critical care hemodynamic monitoring	Analyzes arterial pressure waveform to assess cardiac output	Real‐time blood flow and pressure analysis, non‐invasive monitoring	Edwards Lifesciences	[123]
Mikro‐Cath Pressure Catheter	Minimally invasive hemodynamic monitoring in adult and pediatric patients	Integration with external data acquisition systems; real‐time digital pressure readings	MRI‐compatible catheter with a solid‐state pressure sensor that provides high‐fidelity, real‐time waveform capture in vessels or heart chambers.	Millar, Inc.	[124]
CardioMEMS HF System	Remote hemodynamic monitoring in heart failure patients	Real‐time pulmonary artery pressure for clinician view	Implantable pulmonary artery sensor with battery‐free, passive MEMS	Abbott Laboratories	[125]
Mon‐a‐therm Foley Catheter with Temperature Sensor 400TM	Continuous core temperature monitoring	Real‐time temperature monitoring	Automated readings that provide immediate feedback	Medtronic	[126]
Vis‐Rx Micro‐Imaging Catheter	Cardiovascular imaging	High frequency OCT for vessel imaging	Rotating fiber‐optic probe	Gentuity	[128, 129]
Pantheris Atherectomy Catheter	Peripheral arterial disease treatment	Embedded OCT for guided atherectomy	OCT imaging, precise plaque excision tools	Avinger	[130]
AcuNav Lumos ICE Ultrasound Catheter	Intracardiac imaging	Enhanced imaging resolution with electromagnetic navigation	ICE catheter with high‐resolution ultrasound for cardiac interventions	Siemens Healthineers	[131]
Bravo pH Capsule System	Gastroesophageal reflux disease diagnosis	Esophageal pH monitoring for acid reflux detection	Wireless capsule‐based pH sensor, 96‐h data recording	Medtronic	[134, 135]
Digitrapper pH‐Z Testing System	Gastroesophageal reflux disease diagnosis	Esophageal pH and impedance monitoring to detect acid, weak acid and non‐acid reflux events	Catheter with up to 2 pH sensors and six impedance channels, 48 h data recording	Medtronic	[136, 137]
ZepHr Impedance/pH Reflux Monitoring System	Gastroesophageal reflux disease diagnosis	Combines impedance and pH sensors to detect and categorize all types of reflux events	Omega‐based; multi‐observer validation	Diversatek Healthcare	[138, 139]
Optilume Urethral Drug‐Coated Balloon	Urology (urethral stricture treatment)	Drug‐coated balloon delivers paclitaxel to prevent urethral stricture recurrence	Paclitaxel drug coating, balloon dilation technology	Laborie	[140]

Many companies are working to commercialize advanced catheters that are essential in the surgical treatment of cardiac arrhythmias, particularly during RF or pulsed‐field ablation procedures. These catheters are equipped with sensors that measure contact force, magnetic orientation, or optical tip pressure. The data from these sensors are fed into 3D mapping systems, allowing clinicians to navigate complex heart anatomy with reduced reliance on fluoroscopy. This helps ensure stable contact with tissue and enables the creation of more consistent lesions, which in turn reduces the need for repeat procedures and shortens overall treatment time.

One example is the ThermoCool SmartTouch catheter,^[^ ^117^ ^]^ designed specifically for visualizing and treating cardiac arrhythmias. It includes a precision spring that responds to contact force, as well as a built‐in force sensor and transmitter coil to help ensure accurate positioning.

Similarly, the Map‐iT^[^ ^118^ ^]^ products include a variety of diagnostic electrophysiology catheters that enhance the treatment of complex arrhythmias. These catheters utilize magnetic guidance coupled with integrated feedback for precise mapping, quality signal acquisition, and durability. They are designed for improved maneuverability and performance, offering different configurations to suit both patient and physician.

The TactiCath catheter^[^ ^119^ ^]^ is another example of a commercially available ablation catheter that uses dual Fabry‐Pérot optical sensors embedded in the tip to measure both axial and lateral contact forces with sub‐gram resolution (≈0.3 g). The data is continuously streamed to the EnSite™ Precision mapping platform,^[^ ^120^ ^]^ which can be configured to collect mapping points only when the catheter maintains contact within a specified force window. This ensures more accurate and reproducible lesion formation during ablation. An advancement over the TactiCath catheter, the TactiFlex catheter,^[^ ^121^ ^]^ introduces a laser‐cut flexible tip that can conform to the tissue surface, enhancing stability and allowing for more uniform irrigation during RF energy delivery. Integrated with the EnSite X mapping system,^[^ ^122^ ^]^ it offers real‐time force visualization and deflection indicators that help improve catheter handling and safety. Its flexible design also reduces tip migration, which can otherwise lead to ineffective lesion formation or tissue injury.

Several commercially available catheter systems are also being used for sensing physiological parameters and real‐time monitoring, particularly in surgical and critical care contexts. These systems integrate embedded sensors and algorithm‐driven signal processing to provide a quantitative measure of parameters such as temperature, pressure, and stroke volume every few seconds, allowing clinicians to act immediately before any life‐threatening situation presents itself. The FloTrac® Hemodynamic Monitoring System^[^ ^123^ ^]^ is an example of such a system that provides continuous, minimally invasive hemodynamic monitoring, offering real‐time insights that enable clinicians to make proactive decisions during moderate to high‐risk surgeries and in acute care settings. It uses a validated algorithm that adjusts for patient variability, ensuring accurate and dynamic assessment of stroke volume, cardiac output, and mean arterial pressure. Another widely used tool is the Millar Mikro‐Cath™ Pressure Catheter platform that provides high‐fidelity, real‐time pressure measurements through a solid‐state sensor embedded at the catheter tip. Its small diameter and motion artifact resistance enable accurate monitoring of cardiovascular, respiratory, and compartmental pressures, making it particularly valuable for continuous diagnostics and physiological assessment in both clinical and research environments.^[^ ^124^ ^]^

Similarly, CardioMEMS HF System^[^ ^125^ ^]^ is a remote pulmonary artery pressure monitoring device used for early heart failure management. A small sensor that is permanently implanted in the distal pulmonary artery via a right heart catheterization procedure continuously measures pulmonary artery pressure changes, which indicate fluid retention in the lungs due to worsening heart failure. It provides continuous, wireless, real‐time monitoring of pulmonary artery pressure and transmits data remotely to clinicians. While this device cannot be identified as a smart catheter, it can be considered smart catheter technology through the implanted sensor itself. It shows the potential of these advanced technologies to be combined with catheters.

The Mon‐a‐therm Foley Catheter^[^ ^126^ ^]^ with Temperature Sensor 400TM integrates a Foley catheter with an embedded temperature sensor. This device enables continuous monitoring of a patient's core temperature in critical care environments, facilitating the early detection of temperature fluctuations that may necessitate clinical intervention. By delivering precise, real‐time data, this product can enhance patient care, empowering clinicians to effectively manage temperature‐related conditions.

Real‐time imaging inside blood vessels and heart chambers is now routine during coronary and structural‐heart procedures. Modern smart catheters pack high‐frequency ultrasound probes, sub‐second pull‐back drives, or tiny optical fibers and pair them with software that renders a 3D image on the screen within seconds. This finer visualization of plaque, stents, and soft tissue helps physicians choose the right device size, prepare the lesion properly, and verify therapy success. These tools range from high‐definition ultrasound catheters to OCT‐guided atherectomy systems.

One such example is the OptiCross HD Coronary Imaging Catheter,^[^ ^127^ ^]^ an IVUS catheter designed for high‐definition coronary imaging. It provides enhanced visualization of vessel morphology, aiding in the diagnosis and treatment of coronary artery disease. The catheter features improved deliverability, flexibility, and imaging resolution, allowing for precise assessment of plaque and stent placement.

The Gentuity Vis‐Rx Micro‐Imaging Catheter^[^ ^128^ ^]^ is a high‐frequency OCT‐based device designed for coronary imaging. With a pullback rate fast enough to image 100 mm in 1 s and an axial resolution of ≈10 µm, the catheter allows rapid and detailed visualization of stent deployment and lesion morphology, aiding in making decisions during percutaneous coronary interventions with a single pullback.^[^ ^129^ ^]^ Similarly, Pantheris^[^ ^130^ ^]^ is an OCT image‐guided atherectomy catheter specifically designed for the precise treatment of peripheral artery disease. It is equipped with an onboard imaging fiber that offers real‐time visualization of arterial layers, enabling operators to remove plaque with improved accuracy. The catheter utilizes an apposition balloon to maintain control during the procedure and features a nosecone that effectively captures and eliminates disease.

The AcuNav Lumos 4D intracardiac echocardiography (ICE) ultrasound catheter^[^ ^131^ ^]^ was developed, delivering high‐resolution, real‐time 3D imaging for intracardiac echocardiography. It enhances the precision of complex structural heart procedures by providing superior visualization of heart structures and assisting in device placement. The catheter allows for unobstructed views, which is particularly beneficial in procedures such as tricuspid valve repair or atrial appendage closure. Additionally, it features significant imaging capabilities, including biplane multiplanar reconstruction, for thorough assessments.

Twenty‐four‐hour esophageal pH monitoring is a crucial diagnostic tool for gastroesophageal reflux disease, especially when symptoms persist despite proton pump inhibitor therapy. In such cases, potential causes include ongoing acid reflux, non‐acid reflux, or functional esophageal disorders. Accurately identifying these etiologies is vital for developing effective treatment strategies.^[^ ^132^ ^]^ Traditional pH monitoring effectively detects acid exposure but cannot identify non‐acid reflux events. To overcome this limitation, catheter‐integrated multichannel intraluminal impedance combined with pH monitoring has been developed. This technology enables the detection of both acid and non‐acid reflux and tracks bolus movement throughout the esophagus, providing temporal correlation with symptoms. These features are particularly valuable in diagnosing non‐erosive reflux disease, in which endoscopic findings are normal despite the presence of typical symptoms.^[^ ^133^ ^]^

The Bravo pH Monitoring System^[^ ^134^ ^]^ is a wireless capsule‐based solution that attaches to the esophageal mucosa via endoscopy. It transmits pH data for up to 96 h, offering excellent patient comfort by eliminating the need for a transnasal catheter. However, its function is limited to acid detection and lacks impedance capabilities.^[^ ^135^ ^]^

Another catheter‐based device that integrates pH and impedance sensors is the Digitrapper pH‐Z System.^[^ ^136^ ^]^ It detects acid, weakly acidic, and non‐acid reflux episodes and measures the proximal extent of refluxate. This system is especially useful in assessing patients with persistent symptoms of proton pump inhibitor therapy. Outcome studies have shown that impedance‐pH monitoring identifies additional reflux patterns not detected by pH monitoring alone, although clinical outcomes may not always differ significantly.^[^ ^137^ ^]^

The ZepHr pH and Impedance Monitoring System^[^ ^138^ ^]^ provides combined impedance and pH monitoring and is widely used in both clinical and research settings. Smart catheter systems have significantly enhanced the diagnosis of gastroesophageal reflux and related disorders by integrating impedance technology. This technology enables comprehensive reflux profiling beyond acid detection, and has been validated, demonstrating high technical reliability in reflux detection and symptom correlation.^[^ ^139^ ^]^

Commercial smart catheter systems are also being used for drug delivery and localized, controlled release of therapeutic agents. For example, the Optilume BPH Catheter System^[^ ^140^ ^]^ is a minimally invasive solution for treating benign prostatic hyperplasia (BPH). It combines mechanical dilation with localized delivery of paclitaxel—an antiproliferative drug—to prevent tissue regrowth. The dual‐lobe balloon in the design separates prostatic lobes while delivering the drug, enhancing long‐term symptom relief. This combination has the potential to significantly enhance the effectiveness of BPH treatment.

Together, these innovations underscore the evolution and diversification of the smart catheter market, showcasing the integration of advanced sensor technologies, real‐time data acquisition, and targeted therapeutic capabilities to meet growing clinical demands.

In addition to commercial products, significant public funding initiatives worldwide, especially in the United States and the European Union, are supporting the development of next‐generation smart catheter technologies. In the United States, the federally funded Small Business Innovation Research (SBIR) and Small Business Technology Transfer programs assist entrepreneurs in developing groundbreaking healthcare technologies.^[^ ^141^, ^142^, ^143^ ^]^ The Horizon program and the European Innovation Council serve as primary funding sources in the European Union.

One such project is the UrinControl System, a urinary catheter‐like prosthesis designed to assist individuals with neurogenic bladder dysfunction. The device uses AI to monitor early signs of urosepsis and bladder levels, allowing patients to empty their bladder with the press of a button. The built‐in fail‐safe valve prevents over‐pressurization.^[^ ^144^ ^]^ Another example initiative is an SBIR‐funded project aimed at reducing CAUTIs through the development of a novel anti‐infective coating. This technology integrates zwitterionic moieties to enhance resistance to biofilm formation alongside Gemini‐dicationic moieties that provide sustained antimicrobial activity. Preliminary bench studies have demonstrated a significant decrease in biofilm accumulation. The project's next steps involve in‐vitro testing and incorporation into catheter manufacturing processes, with the broader objective of enabling applications across a range of implantable medical devices.^[^ ^145^ ^]^ Furthermore, a smart diagnostic mapping catheter has been developed to enhance the precision of AF ablation by accurately identifying AF sources. The device features a 24‐electrode configuration arranged in a 2D matrix of bipolar pairs, paired with an advanced analysis algorithm validated in AF patients. This technology aims to improve targeted ablation by leveraging data‐driven localization of AF sources. Current efforts focus on the fabrication and evaluation of the catheter to optimize its clinical performance, ultimately improving mapping accuracy and procedural outcomes in AF treatment.^[^ ^146^ ^]^

In Europe, a project coordinated by Philips Electronics Nederland BV and supported by the Electronic Components and Systems for European Leadership Joint Undertaking in collaboration with the Horizon 2020 program aims to revolutionize catheter manufacturing by integrating advanced technologies and streamlining supply chains and to establish a pilot line for next‐generation smart catheters built on open technology platforms to foster industry‐wide collaboration.^[^ ^147^ ^]^ Key innovations include miniaturization, integration of analog‐to‐digital converters at the catheter tip, incorporation of ultrasound MEMS, and advanced component encapsulation. By optimizing workflows in catheter laboratories, this project seeks to create a more intuitive environment for surgeons, reducing errors and improving procedural efficiency. Additionally, the initiative will bolster Europe's research and development and manufacturing capabilities, supporting broader advancements in bioelectronic implants for healthcare.^[^ ^147^, ^148^ ^]^

LUMA Vision Limited, based in Germany and Ireland, is developing a novel smart catheter system named Verafeye, to improve the treatment of AF.^[^ ^149^ ^]^ It provides real‐time 4D tissue analysis and navigation during ablation procedures, enabling surgeons to monitor and adjust their techniques based on objective data rather than relying solely on experience. This catheter‐based imaging system leverages advanced imaging and analytics to enhance the precision of ablation procedures, representing a significant advancement in evidence‐based cardiac care.

Lastly, a project coordinated by Amferia AB focused on creating a next‐generation urinary catheter designed to prevent CAUTIs.^[^ ^150^ ^]^ It utilizes a patented amphiphilic antibacterial material developed by Amferia, which kills antibiotic‐resistant bacteria on contact while remaining safe for human cells. A significant challenge faced by the project was transforming this paste‐like material into a tubular structure suitable for catheters. To address this, they modified the traditional polymer extrusion device to accommodate the material's unique properties. This innovation aims to prevent bacterial attachment and biofilm formation, ultimately reducing infection rates, lowering antibiotic dependence, and shortening hospital stays. By doing so, it seeks to improve patient outcomes and address the growing threat of antibiotic resistance in healthcare settings.

Market entry for smart catheters, or any medical device, is driven by a set of regulatory steps in the United States. Devices are classified by risk into Class I (low risk), Class II (moderate risk), and Class III (high risk). Many Class I devices are exempt from premarket submissions and rely on general controls. Most Class II devices require a Premarket Notification (510(k)) demonstrating substantial equivalence to a legally marketed predicate. Class III devices that sustain or support life or present heightened risk generally require Premarket Approval. Regardless of class, manufacturers must meet general controls, including establishment of registration, device listing, quality‐system requirements (21 CFR 820 / QMSR), and labeling regulations. For novel, moderate‐risk devices without a suitable predicate, the De Novo pathway, which is a risk‐based route for first‐of‐a‐kind devices that lets the U.S. Food and Drug Administration create a new Class I or II device type, may be appropriate; clinical investigations, when needed, proceed under an investigational device exemption.^[^ ^151^ ^]^ In parallel, the manufacturing scale should be defined under a documented quality‐management system with supplier controls and process validation to maintain yield on curved, multi‐material assemblies.^[^ ^152^ ^]^ The sterilization method should be selected and validated, shelf life should be established, and any calibration of drift or material/coating changes should be quantified using pre‐/post‐sterilization metrology.^[^ ^153^ ^]^ Human‐factors/usability engineering should begin early (tasks, users, environments), with findings cycled back into design so that setup, navigation, and training align with clinical workflow.^[^ ^154^ ^]^ For connected or console‐integrated devices, interoperability and cybersecurity should be addressed within design controls and in the premarket submission package (e.g., threat modeling, update policy, software bill of materials, logging).^[^ ^155^ ^]^ Finally, a reprocessing strategy (single‐use or validated reuse) should be defined, and test‐supported instructions for use should be provided for cleaning, disinfection, and sterilization, as applicable. Addressing these elements alongside clinical evidence substantially reduces late‐stage barriers to adoption.^[^ ^156^ ^]^

## 6 Challenges and Future Outlook

Smart catheter technologies have emerged as transformative medical tools, significantly advancing diagnostics, therapeutics, and minimally invasive interventions. Despite notable progress in integrating sophisticated sensing systems, biocompatible materials, and robotic functionalities, several substantial challenges continue to restrict their broader adoption. Addressing these challenges—which span materials science, sensor integration, manufacturing scalability, clinical translation, and regulatory acceptance paramount to fully leverage the clinical potential of smart catheters.

A comparative analysis of the properties of smart and traditional catheters is summarized in Table S1 (Supporting Information). Addressing the challenges of biocompatibility, sensor integration, infection control, structural durability, cost, and regulatory complexity will be crucial for the future progress of smart catheter technologies. As interdisciplinary research continues to address these barriers, the next generation of smart catheters promises transformative impacts on healthcare, enabling highly precise diagnostics, targeted therapeutics, and minimally invasive interventions, ultimately improving patient outcomes and reshaping the landscape of personalized medicine.

A primary challenge in advancing smart catheter technology involves achieving reliable long‐term biocompatibility. Prolonged exposure of catheter materials to physiological environments inevitably triggers responses such as protein adsorption, thrombus formation, and microbial colonization, leading to biofilm formation.^[^ ^157^ ^]^ Biofilms remain a significant source of catheter‐associated infections, severely impacting patient health and healthcare economics.^[^ ^158^ ^]^ Although strategies such as nanoparticle‐infused coatings,^[^ ^159^ ^]^ silver‐based antimicrobial materials,^[^ ^160^ ^]^ graphene oxide composites,^[^ ^161^ ^]^ and zwitterionic polymers^[^ ^162^ ^]^ have shown promise in mitigating these issues, they typically lack sustained antimicrobial efficacy throughout extended catheter use. Therefore, future research must emphasize innovative materials—including stimuli‐responsive hydrogels, shape‐memory polymers, and multifunctional nanocoatings—to achieve durable, long‐lasting antifouling and antimicrobial performance. Future advances are expected to incorporate self‐regenerating antimicrobial coatings, bio‐inspired surface textures, and microfluidic approaches designed explicitly to disrupt microbial colonization and sustain antimicrobial efficacy over extended periods.

Sensor integration within catheters presents an additional challenge. Incorporating advanced electronic components and multimodal sensors capable of simultaneous detection of biochemical and biophysical parameters requires careful engineering to ensure catheter flexibility, reliability, and performance. The integration of wireless communication for real‐time data transmission introduces further complexity due to signal attenuation by biological tissues, sensor miniaturization constraints, and power management issues. To address these constraints, ongoing research explores flexible electronics,^[^ ^109^ ^]^ innovative power transfer methods,^[^ ^163^ ^]^ and advanced encapsulation techniques^[^ ^164^ ^]^ that enhance sensor stability and reliability under physiological conditions.

Structural durability and material limitations also significantly constrain the clinical performance of smart catheters. Traditional catheter materials, such as silicone or polyurethane, frequently experience degradation, mechanical failure, and compromised flexibility after prolonged use and repeated sterilization processes.^[^ ^165^ ^]^ New‐generation flexible materials—including stretchable electronics, nanocomposite‐reinforced elastomers, and bio‐responsive polymers—are under active development to improve resilience under mechanical stress, maintain sensor integrity, and extend device lifespans. Such advancements are crucial for enhancing catheter safety, patient comfort, and overall clinical effectiveness.

The high cost and limited scalability of smart catheter manufacturing processes impede broader clinical adoption. Production methods rely on specialized and costly fabrication steps, limiting widespread accessibility. Promising scalable techniques, such as roll‐to‐roll printing,^[^ ^166^ ^]^ laser‐induced graphene patterning,^[^ ^167^ ^]^ inkjet printing,^[^ ^168^ ^]^ and additive manufacturing,^[^ ^169^ ^]^ present substantial opportunities to streamline production, reduce costs, and maintain high quality. Modular designs and standardized fabrication protocols offer additional avenues for developing reproducible, cost‐effective catheter devices, thus facilitating their translation from laboratory prototypes to mass‐produced clinical tools. The continued evolution of additive manufacturing, along with high‐volume fabrication techniques borrowed from flexible electronics production, will drive smart catheter innovation from laboratory prototypes to affordable clinical products. Achieving manufacturing scalability is essential for broadening market accessibility and widespread clinical integration.

Regulatory and clinical workflow challenges further complicate the translation of smart catheter technologies into clinical practice.^[^ ^170^ ^]^ The interdisciplinary nature of smart catheters, integrating electronic, biomedical, and therapeutic elements, necessitates navigating rigorous regulatory processes. Ensuring device safety, efficacy, and biocompatibility demands clear performance benchmarks, standardized testing methods, and comprehensive clinical validation. Further, adoption into practical use requires clinicians to realize added value, become proficient in use, and ultimately demonstrate consistent improved outcomes resulting from integration into standard practice. Collaborative efforts among academia, industry, and regulatory authorities are crucial to streamline approval pathways, standardize evaluation criteria, and produce robust clinical evidence supporting the advantages of smart catheters over traditional devices. Therefore, successful translation ultimately hinges on interdisciplinary convergence, across materials, electronics, software/AI, clinical practice, and regulation, co‐designed around shared benchmarks and aligned workflows from prototype to bedside.

As smart catheters evolve toward network‐connected, AI‐assisted, and closed‐loop operation, security‐by‐design and ethics‐by‐design must progress in parallel with sensing and control. Practically, this includes authenticated and encrypted telemetry, signed firmware, and formal threat modeling appropriate for safety‐critical devices, privacy‐preserving learning (on‐device preprocessing, federated learning with secure aggregation) to enable multi‐site model improvement while minimizing exposure of protected health information, and prospective safeguards against algorithmic bias and performance drift via transparent model provenance, representative validation, and post‐deployment monitoring with human‐in‐the‐loop oversight commensurate with clinical risk. Aligning these practices with contemporary medical‐device cybersecurity and trustworthy‐AI guidance will be essential to maintain patient trust, meet regulatory expectations, and safely scale intelligent catheter platforms.^[^ ^171^, ^172^ ^]^

The future trajectory of smart catheter technology is diverse and highly promising. Advances will likely feature the increasing integration of new technologies from materials science, AI, robotics, multimodal sensing, and advanced manufacturing. AI‐driven navigation algorithms and robotic‐assisted catheter systems are expected to significantly enhance precision during minimally invasive interventions, allowing adaptive real‐time control tailored to patient‐specific anatomical complexities. Multimodal sensor arrays coupled with advanced data analytics will elevate diagnostic capabilities, enabling continuous patient monitoring, early complication detection, and intelligent therapeutic interventions.

Next‐generation therapeutic catheter designs will advance significantly beyond current drug‐eluting capabilities. Innovations such as microneedle‐equipped balloon catheters^[^ ^17^ ^]^ and biodegradable drug‐eluting^[^ ^173^ ^]^ platforms promise precise, localized therapy delivery. Future catheters may incorporate advanced biological therapeutics, including exosomes, RNA therapies, and therapeutic proteins, and implantable precision sensors, facilitating personalized and biologically targeted interventions. These technologies represent a transformative shift toward integrated, closed‐loop therapeutic systems capable of autonomous sensing, decision‐making, and targeted drug delivery. Next‐generation therapeutic catheter designs will advance significantly beyond current drug‐eluting capabilities. Innovations such as microneedle‐equipped balloon catheters^[^ ^17^ ^]^ and biodegradable drug‐eluting^[^ ^173^ ^]^ platforms promise precise, localized therapy delivery. Future catheters may incorporate advanced biological therapeutics, including exosomes, RNA therapies, and therapeutic proteins, facilitating personalized and biologically targeted interventions. These technologies represent a transformative shift toward integrated, closed‐loop therapeutic systems capable of autonomous sensing, decision‐making, and targeted drug delivery.

## 7 Conclusion

Catheters have evolved beyond their traditional roles in access and drainage to become multifunctional platforms for in vivo diagnosis, monitoring, and therapy. Leveraging advancements in sensing technologies, biomaterials, wireless communication, and robotic navigation, smart catheters enable real‐time health monitoring, early disease detection, and enhanced precision in minimally invasive procedures. Moreover, they address critical limitations of conventional catheters, such as infection risk, occlusion, and long‐term tissue damage, offering safer and more effective clinical solutions. In this review, we examined the wide range of smart catheter designs – from single‐sensor tools to complex, multimodal systems – and the ways they are changing clinical practices. Nonetheless, important challenges remain, especially when making these devices safer for long‐term use, cost‐effective to produce, and easy to bring into everyday hospital workflows. Even with these remaining challenges, the progress to date points to a future where smart catheters could play a much bigger role in healthcare, making treatments safer or more efficient and helping move medicine to real‐time, personalized care that fits patient needs moment‐by‐moment.

## Conflict of Interest

The authors declare no conflict of interest.

## Author Contributions

A.Y.T., S.A., and M.O.A., contributed equally to this work. A.Y.T., S.A., and M.O.A. conducted the literature review and curated data from the cited literature studies, drafted the initial manuscript, and prepared all figures and tables. M.G. and D.C.L. contributed to drafting the initial manuscript. F.S.L., G.T.L., and R.O. provided project oversight, critical revisions of the manuscript, and writing support. H.K. provided critical revisions to the manuscript and contributed to the section on commercially available catheters and the tables. M.J.M. and J.K. provided critical revisions of the manuscript and oversight of the sensor‐integrated catheters and their fabrication technologies. F.S.L., M.J.M., J.B., and H.C.K. contributed to the funding acquisition. H.C.K. conceptualized the idea, provided project oversight, made critical revisions to the manuscript, and supervised the project. The manuscript was written through the contributions of all authors. All authors have approved the final version of the manuscript.

## Supporting information



Supporting Information
