# Supplementary material for: Smart Catheters for Diagnosis, Monitoring, and Therapy
Source: Adv Healthc Mater. 2025 Nov 17;15(13):e03913. doi: 10.1002/adhm.202503913 (PMC12721224; doi:10.1002/adhm.202503913)
Supplement: Supplementary file 1 — Supporting Information [file ADHM-15-0-s001.docx]

**Supplementary Information**

**Smart Catheters for Diagnosis, Monitoring, and Therapy**

Azra Yaprak Tarman^‡,1,2^, Samiha Ahmed^‡,1,2^, Majed Othman Althumayri^‡,1,2,3^, Megan Guy^2,4^, Darlenne Chavez Lugo^1,2^, Frances S. Ligler^1^, George T. Ligler^4^, Rahmi Oklu^5^, Hiroshi Kawahira^6,7^, Michael J. McShane^1,2,8^, Jun Kameoka^9^, Jonathan Bova^10^, Hatice Ceylan Koydemir^1,2,*^

^1^Department of Biomedical Engineering, Texas A&M University, College Station, Texas, 77843, United States

^2^Center for Remote Health Technologies and Systems, Texas A&M Engineering Experiment Station, College Station, Texas, 77843, United States

^3^Department of Medical Equipment Technology, College of Applied Medical Sciences, Majmaah University, Al Majmaah 11952, Saudi Arabia

^4^Department of Multidisciplinary Engineering, Texas A&M University, College Station, Texas, 77840, United States

^5^Laboratory for Patient Inspired Engineering, Mayo Clinic, Phoenix, Arizona, 85054, United States

^6^Department of Surgery, Division of Gastroenterological, General and Transplant Surgery, Jichi Medical University, 3311-1, Yakushiji, Shimotsuke-shi, Tochigi, 329-0498, Japan

^7^Medical Simulation Center, Jichi Medical University, 3311-1, Yakushiji, Shimotsuke-shi, Tochigi, 329-0498, Japan

^8^Department of Materials Science and Engineering, Texas A&M University, College Station, Texas 77843, United States

^9^Graduate School of Fundamental Science and Engineering, Information, Production and System Research, Waseda University, Kitakyushu, Fukuoka, 808-0135, Japan

^10^Comparative Medicine Program, Division of Research, Texas A&M University, College Station, TX 77843, United States

*Corresponding author

‡These authors contributed equally.

**LIST OF ABBREVIATIONS**

| 2D | Two dimensional |
| --- | --- |
| 3D | Three dimensional |
| 4D | Four dimensional |
| AF | Atrial fibrillation |
| BPH | Benign prostatic hyperplasia |
| CAGR | Compound annual growth rate |
| CAUTI | Catheter-associated urinary tract infection |
| CRP | C-reactive protein |
| CVC | Central venous catheters |
| CTC | Circulating tumor cells |
| DEB | Drug-eluting balloon |
| ECG | Electrocardiogram |
| FBG | Fiber Bragg grating |
| FSCR | Ferromagnetic soft catheter |
| ICE | Intracardiac echocardiography |
| ISAC | Integrated self-assembled catheter |
| IVUS | Intravascular ultrasound |
| MEMS | Micro-electromechanical systems |
| MIP | Molecularly imprinted polymer |
| MNDEB | Microneedle drug-eluting balloon |
| MN | Microneedle |
| MRI | Magnetic resonance ımaging |
| MSRC | Miniaturized soft robotic catheter |
| NFC | Near-field communication |
| NIRF | Near-infrared fluorescence |
| OCT | Optical coherence tomography |
| OSA | Obstructive sleep apnea |
| PDMS | Polydimethylsiloxane |
| PFA | Pulsatile field ablation |
| PVDF | Polyvinylidene-fluoride |
| RF | Radiofrequency |
| SBR | Small business innovation research |
| SEM | Scanning electron microscopy |
| TrFE | Trifluoroethane |
| UV | Ultraviolet |
| VSSM | Variable stiffness stabilizing mechanism |
| VST | Variable stiffness thread |

**Table S1**. Side-by-side comparison of smart versus traditional catheters highlighting key differences in functionality and performance.

| **Property** | **Smart Catheters** | **Traditional Catheters** | **Reference** |
| --- | --- | --- | --- |
| **Navigation** | Uses robotic or AI-driven systems for precise navigation in complex anatomies​. | Relies on manual operator skill; limited precision in tortuous pathways. | ^[1,2]^ |
| **Sensing Capabilities** | Integrated sensors provide real-time feedback on pressure, force, and biomarkers. | No integrated sensing; depends on external imaging and tactile feedback. | ^[3,4]^ |
| **Therapeutic Delivery** | Enables targeted drug delivery through integrated systems. | No therapeutic delivery mechanisms; uses separate tools. | ^[5,6]^ |
| **Durability** | Advanced coatings reduce biofouling and increase lifespan. | Susceptible to biofouling and degradation during extended use. | ^[7–9]^ |
| **Patient Outcomes** | Enhanced precision lowers procedural risks, reduces trauma, and improves recovery times. | Greater risk of complications due to limited adaptability and manual precision. | ^[10,11]^ |
| **Ease of Use** | Requires specialized training; semi-autonomous systems can reduce operator workload. | Simpler to use but requires high operator skill for complex procedures. | ^[12,13]^ |
| **Energy Requirements** | Requires energy for sensors, actuators, and wireless communications. | No power requirements; fully manual operation. | ^[14]^ |
| **Cost Efficiency** | Higher upfront cost but reduces hospital stay and complications, offering long-term cost savings. | Lower initial costs but higher long-term costs due to procedural complications. | ^[15,16]^ |

**Table S2.** Comparison of fabrication and integration methods for smart catheters with advantages, disadvantages, and their representative applications.

| **Fabrication Methods** | **Advantages** | **Disadvantages** | **Applications** | **References** |
| --- | --- | --- | --- | --- |
| Photolithography/ Soft lithography | High precision; wafer-scale repeatability; high throughput | Strain due to conformal wrapping; curvature-induced misalignment | Patterned electrodes/sensors;  temperature/pressure arrays | ^[17–21]^ |
| Transfer printing | Heterogeneous integration; supports ultrathin sensors | Alignment and adhesion complexity | Lift-off of prefabricated microstructures to curved elastomers/balloons | ^[22–26]^ |
| Direct laser micromachining | Can be printed directly on 3D structures; rapid iterations possible; no mask required | High equipment cost; thermal or ablation artifacts; limited throughput | Direct patterning/engraving microchannels on a curved surface | ^[26,27]^ |
| Plasma bonding/multilayer laminations | Strong adhesion; biocompatible interfaces | Critical process control | Robust assembly of elastomers with embedded traces | ^[26]^ |

**Table S3.** Comparison of major materials used in smart catheters, showing advantages, disadvantages, and their applications.

| **Materials** | **Advantages** | **Disadvantages** | **Applications** | **References** |
| --- | --- | --- | --- | --- |
| Shape-memory polymers/conductive shape-memory polymers | Variable stiffness; large stiffness control range; lightweight | Heat-management requirements; fatigue under repeated stiffness cycling | Cardiac navigation, temporary shape lock | ^[28,29]^ |
| Low-melting-point alloys | Large modulus change; improved flexibility during tortuous navigation | Thermal-control requirements | Temporary support during catheter deployment; reversible rigid-to-flexible transition | ^[30]^ |
| Liquid metal-polymer conductors | High stretchability; self-healing; low resistance | Need for robust encapsulation to prevent leakage; potential cytotoxicity on exposure | Stretchable electrodes; ablation catheters | ^[31]^ |
| Graphene/CNT nanomaterials | High surface area; suitability for sensor integration | Long-term biocompatibility issues | Flexible electronics | ^[32]^ |
| Antifouling/antimicrobial coatings (e.g., Ag nanoparticles) | Dual action (anti-adhesion and antimicrobial activity); efficacy under flow | Requirement to maintain effective silver dose; potential durability issues | Urinary & long-dwell catheters | ^[33–36]^ |
| Thermoresponsive hydrogel coatings | Conformal coverage; soft mechanics; low cytotoxicity | Hydration state drift; mechanical wear | Real-time temperature sensing and monitoring | ^[37]^ |

**Table S4.** Comparison of data acquisition and communication options for catheter systems in terms of advantages, disadvantages, and their applications.

| **Modalities** | **Advantages** | **Disadvantages** | **Applications** | **References** |
| --- | --- | --- | --- | --- |
| NFC | Battery-free operation; simple user interface; suitable for short, periodic reads | Very short range; Sensitive to orientation and distance; limited throughput | Wireless, periodic sensing | ^[38]^ |
| Bluetooth | Continuous data streaming; long-range data transfer; robust security | High power requirements | Real-time oximetry, multi-sensor streaming | ^[39]^ |
| On-device microcontroller processing | Reduced raw bandwidth; source-level filtering; support for closed-loop operation | Firmware complexity | Any continuous sensing needing low latency | ^[23]^ |

**Table S5.** Comparison of navigation and actuation technologies in smart catheters, showing advantages, disadvantages, and their representative applications.

| **Technologies** | **Advantages** | **Disadvantages** | **Applications** | **References** |
| --- | --- | --- | --- | --- |
| Magnetic actuation (external magnetic fields and ferromagnetic tips) | No on-tip hydraulics; remote control; high precision; fast | Requires magnetic field generators; limited torque in tortuous paths | Neurovascular navigation; targeted manipulation | ^[32,40–42]^ |
| Shape-memory alloy tips | High force in a small footprint | Heating latency; fatigue | Simple steerable tips; various shape settings | ^[29]^ |
| Hydraulic/pneumatic soft actuators | Gentle interaction; large strokes; higher degree of freedom | Tubing/control overhead (long microtubes and external pumps required); hysteresis | Navigation through beating organs like heart; vascular stabilization | ^[29,43]^ |
| Variable stiffness soft robotic technology | Navigation in a flexible state; rigid support during therapy | Thermal management requirement; moderate speed | Sub-millimeter access, coil deployment support | ^[29,30,43]^ |
| Flow-driven navigation (with magnetic assist) | Exploits hemodynamics; reduced external pushing | Path dependence; control complexity | Ultra-flexible probes in the brain vasculature | ^[40]^ |
| Ferromagnetic soft robotic technology | Enables minimally invasive deposition; Precise localized motion | Complex materials; hard to maintain sterility; limited reuse | Tissue repair/ in situ bioprinting | ^[42]^ |

**References:**

[1] A. Ali, A. Sakes, E. A. Arkenbout, P. Henselmans, R. van Starkenburg, T. Szili-Torok, P. Breedveld, *Proc Inst Mech Eng H* **2019**, *233*, 1207.

[2] C. Gianni, A. Natale, R. P. Horton, in *Catheter Ablation of Cardiac Arrhythmias (Fourth Edition)* (Eds: S. K. S. Huang, J. M. Miller), Elsevier, Philadelphia, **2019**, pp. 117-125.e2.

[3] A. Ramadani, M. Bui, T. Wendler, H. Schunkert, P. Ewert, N. Navab, *Medical Image Analysis* **2022**, *82*, 102584.

[4] J. Park, B. Seo, Y. Jeong, I. Park, *Advanced Science* **2024**, *11*, 2307427.

[5] K. Stærk, J. S. Hjelmager, M. Alm, P. Thomsen, T. E. Andersen, *Urologic Oncology: Seminars and Original Investigations* **2022**, *40*, 409.e19.

[6] V. Iacovacci, L. Ricotti, E. Sinibaldi, G. Signore, F. Vistoli, A. Menciassi, *Adv Sci (Weinh)* **2018**, *5*, 1800807.

[7] N. Bouhrour, P. H. Nibbering, F. Bendali, *Pathogens* **2024**, *13*, 393.

[8] S. Moayedi, W. Xia, L. Lundergan, H. Yuan, J. Xu, *Langmuir* **2024**, *40*, 23125.

[9] B. W. Trautner, R. O. Darouiche, *Am J Infect Control* **2004**, *32*, 177.

[10] V. Gauhar, D. Castellani, J. Y.-C. Teoh, C. Nedbal, G. Chiacchio, A. T. Gabrielson, F. L. Heldwein, M. L. Wroclawski, J. de la Rosette, R. Donalisio da Silva, et al., *J Clin Med* **2022**, *11*, 4463.

[11] ProPlate, *ProPlate®* **2024**.

[12] C. V. Riga, C. D. Bicknell, R. Sidhu, F. Cochennec, P. Normahani, P. Chadha, E. Kashef, M. Hamady, N. J. W. Cheshire, *European Journal of Vascular and Endovascular Surgery* **2011**, *42*, 531.

[13] R. Beyar, J. Davies, C. Cook, D. Dudek, P. Cummins, N. Bruining, *EuroIntervention* **2021**, *17*, 537.

[14] C. Li, P.-M. Wu, J. A. Hartings, Z. Wu, C. H. Ahn, D. LeDoux, L. A. Shutter, R. K. Narayan, *Applied Physics Letters* **2011**, *99*, 233705.

[15] “Expert discusses cost effectiveness of reusable catheters,” can be found under https://www.urologytimes.com/view/expert-discusses-cost-effectiveness-of-reusable-catheters, **2024**.

[16] S. M. Group, “The Journey Toward Intelligent Catheters,” can be found under https://www.medicaldesignbriefs.com/component/content/article/45304-the-journey-toward-intelligent-catheters, **2022**.

[17] Y. Hasegawa, H. Kawaoka, Y. Mitsunari, M. Matsushima, T. Kawabe, M. Shikida, *Microsyst Technol* **2018**, *24*, 3455.

[18] B. Gil, B. Li, A. Gao, G.-Z. Yang, *ACS Appl. Electron. Mater.* **2020**, *2*, 2669.

[19] W. Zhang, L. Zhang, Y. Liao, H. Cheng, *Int. J. Extrem. Manuf.* **2021**, *3*, 042001.

[20] C. Li, P.-M. Wu, Z. Wu, C. H. Ahn, D. LeDoux, L. A. Shutter, J. A. Hartings, R. K. Narayan, *Biomed Microdevices* **2012**, *14*, 109.

[21] B. Alekya, V. S. N. Sitaramgupta V, B. S. Arjun, V. Bhushan, K. Abishek, S. Rao, Y. Kim, H. J. Pandya, *J. Micromech. Microeng.* **2021**, *31*, 055007.

[22] X. Shen, E. Jia, Y. Huang, D. Ge, Z. Sun, Z. Yang, P. Zhang, Y. Chen, X. Feng, *Science Advances* **2024**, *10*, eadq5822.

[23] M. Han, L. Chen, K. Aras, C. Liang, X. Chen, H. Zhao, K. Li, N. R. Faye, B. Sun, J.-H. Kim, et al., *Nat Biomed Eng* **2020**, *4*, 997.

[24] X. Ji, P. Zhou, L. Zhong, A. Xu, A. C. O. Tsang, P. K. L. Chan, *Advanced Science* **2018**, *5*, 1701053.

[25] H.-J. Chung, M. S. Sulkin, J.-S. Kim, C. Goudeseune, H.-Y. Chao, J. W. Song, S. Y. Yang, Y.-Y. Hsu, R. Ghaffari, I. R. Efimov, et al., *Advanced Healthcare Materials* **2014**, *3*, 59.

[26] H. Yi, H. Kim, K. R. Kim, J. H. Kim, J. Kim, H. Lee, S. S. Grewal, W. D. Freeman, W.-H. Yeo, *Biosensors and Bioelectronics* **2024**, *255*, 116267.

[27] J. Shang, X. Ma, P. Zou, C. Huang, Z. Lao, J. Wang, T. Jiang, Y. Fu, J. Li, S. Zhang, et al., *Nat Commun* **2025**, *16*, 287.

[28] Y. Piskarev, J. Shintake, C. Chautems, J. Lussi, Q. Boehler, B. J. Nelson, D. Floreano, *Advanced Functional Materials* **2022**, *32*, 2107662.

[29] C. C. Nguyen, M. T. Thai, T. T. Hoang, J. Davies, P. T. Phan, K. Zhu, L. Wu, M. A. Brodie, D. Tsai, Q. P. Ha, et al., *Sensors and Actuators A: Physical* **2023**, *357*, 114380.

[30] J. Lussi, M. Mattmann, S. Sevim, F. Grigis, C. De Marco, C. Chautems, S. Pané, J. Puigmartí-Luis, Q. Boehler, B. J. Nelson, *Advanced Science* **2021**, *8*, 2101290.

[31] R. Li, J. Wang, X. Zhao, Z. Liu, P. Jia, Y. Liu, G. Lin, H. Xu, J. Xiong, *Biosensors and Bioelectronics* **2025**, *270*, 116977.

[32] P. I. Baburova, D. V. Kladko, A. Lokteva, A. Pozhitkova, V. Rumyantceva, V. Rumyantceva, I. V. Pankov, S. Taskaev, V. V. Vinogradov, *ACS Nano* **2023**, *17*, 20925.

[33] D.-S. Won, H. Lee, Y. Park, M. Chae, Y.-C. Kim, B. Lim, M.-H. Kang, M.-R. Ok, H.-D. Jung, J.-H. Park, *Advanced Healthcare Materials* **2024**, *13*, 2401700.

[34] A. Puertas-Segura, A. G. Morena, S. Pérez Rafael, K. Ivanova, I. Ivanov, K. Todorova, P. Dimitrov, G. Ciardelli, T. Tzanov, *ACS Appl. Mater. Interfaces* **2024**, *16*, 39129.

[35] S. Dai, Y. Gao, L. Duan, *Journal of Applied Polymer Science* **2023**, *140*, e53701.

[36] P. Singha, J. Locklin, H. Handa, *Acta Biomaterialia* **2017**, *50*, 20.

[37] Y. Li, D. Li, J. Wang, T. Ye, Q. Li, L. Li, R. Gao, Y. Wang, J. Ren, F. Li, et al., *Advanced Functional Materials* **2024**, *34*, 2310260.

[38] B. Gil, H. Ip, P. Kassanos, B. Lo, G.-Z. Yang, S. Anastasova, *Materials Today Bio* **2022**, *15*, 100298.

[39] W. Lu, W. Bai, H. Zhang, C. Xu, A. M. Chiarelli, A. Vázquez-Guardado, Z. Xie, H. Shen, K. Nandoliya, H. Zhao, et al., *Science Advances* **2021**, *7*, eabe0579.

[40] L. Pancaldi, P. Dirix, A. Fanelli, A. M. Lima, N. Stergiopulos, P. J. Mosimann, D. Ghezzi, M. S. Sakar, *Nat Commun* **2020**, *11*, 6356.

[41] Q. Yang, Á. Enríquez, D. Devathasan, C. A. Thompson, D. Nayee, R. Harris, D. Satoski, B. Obeng-Gyasi, A. Lee, R. T. Bentley, et al., *Nat Commun* **2022**, *13*, 520.

[42] C. Zhou, Y. Yang, J. Wang, Q. Wu, Z. Gu, Y. Zhou, X. Liu, Y. Yang, H. Tang, Q. Ling, et al., *Nat Commun* **2021**, *12*, 5072.

[43] T. Gopesh, J. H. Wen, D. Santiago-Dieppa, B. Yan, J. Scott Pannell, A. Khalessi, A. Norbash, J. Friend, *Sci Robot* **2021**, *6*, eabf0601.
